# Supplementary material for: Large-Scale Profiling of Kinase Dependencies in Cancer Cell Lines
Source: Cell Rep. 2016 Mar 3;14(10):2490–501. doi: 10.1016/j.celrep.2016.02.023 (PMC4802229; doi:10.1016/j.celrep.2016.02.023)
Supplement: Document S1. Supplemental Experimental Procedures and Figures S1–S5 [file mmc1.pdf]

Cell Reports, Volume *14*

Supplemental Information

Large-Scale Profiling of Kinase Dependencies in Cancer Cell Lines

James Campbell, Colm J. Ryan, Rachel Brough, Ilirjana Bajrami, Helen N. Pemberton, Irene Y. Chong, Sara Costa-Cabral, Jessica Frankum, Aditi Gulati, Harriet Holme, Rowan Miller, Sophie Postel-Vinay, Rumana Rafiq, Wenbin Wei, Chris T. Williamson, David A. Quigley, Joe Tym, Bissan Al-Lazikani, Timothy Fenton, Rachael Natrajan, Sandra J. Strauss, Alan Ashworth, and Christopher J. Lord

SUPPLEMENTAL FIGURES

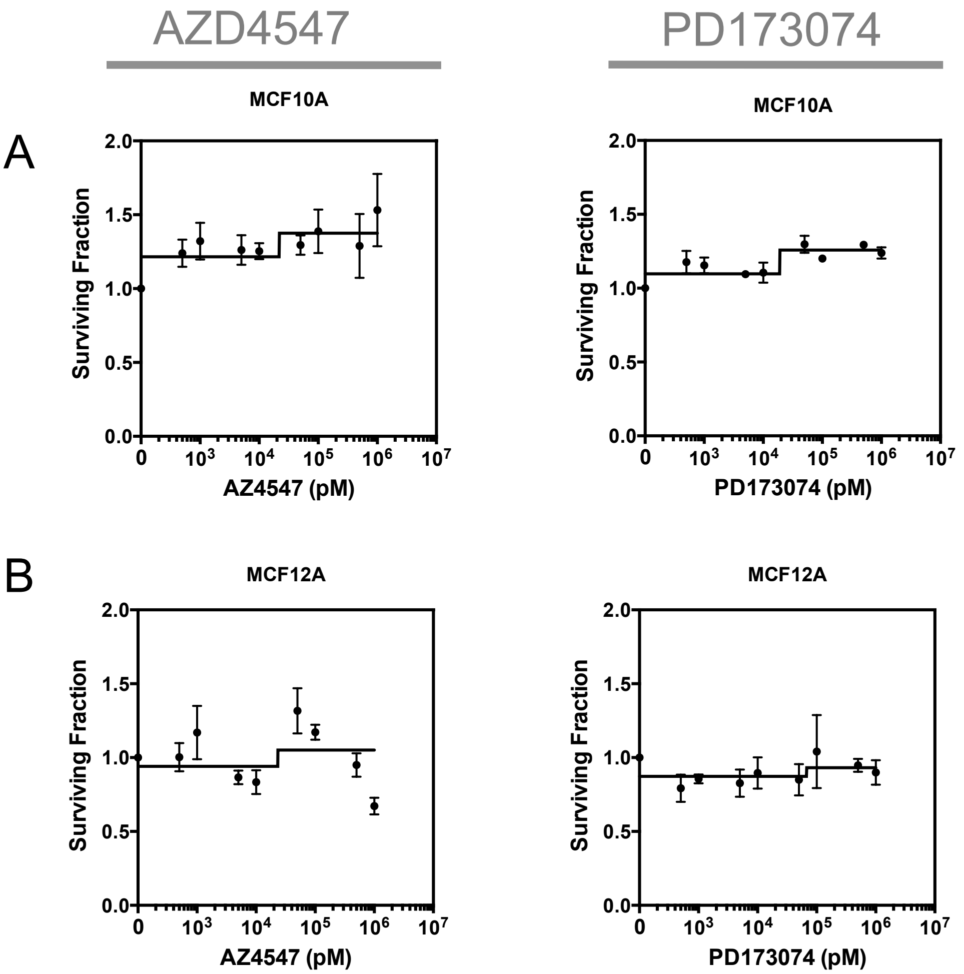

Figure S1. Response to two small molecule FGFR inhibitors (AZD4547 and PD173074) in non-tumour derived epithelial cell lines. (Related to Figure 2). A) Response of the normal epithelium-derived cell line MCF10A to eight concentrations of AZD4547 and PD173074. B) Response of the normal epithelium-derived cell line MCF12A to eight concentrations of AZD4547. Error bars represent standard error of the mean from three independent experiments.

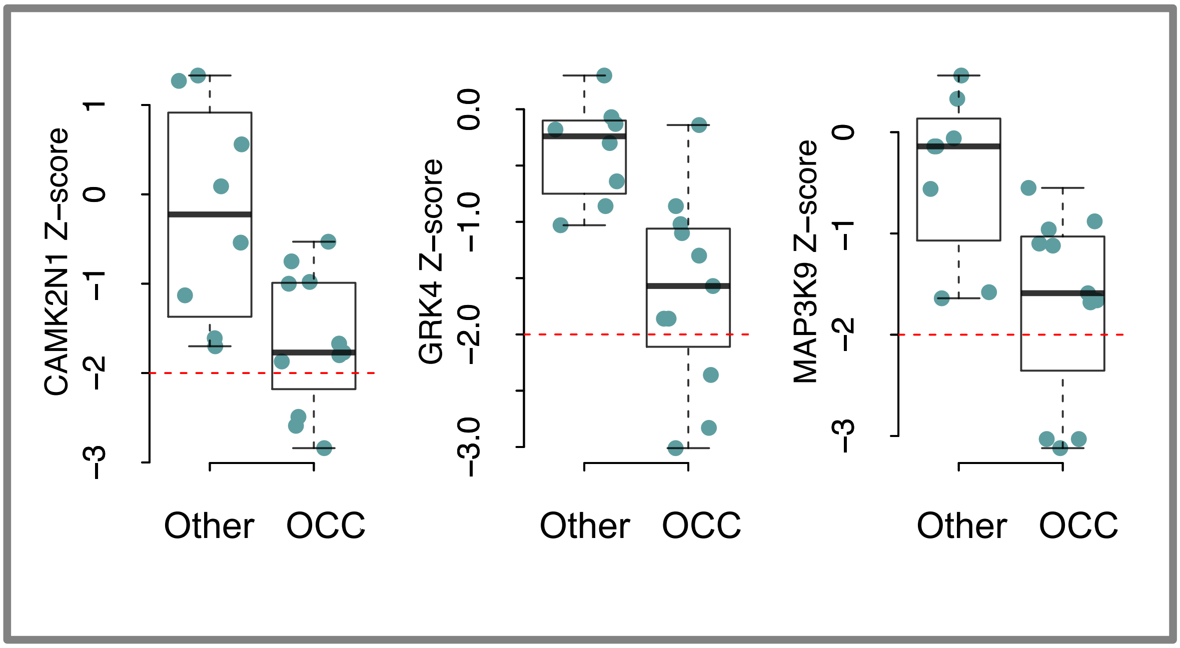

Figure S2. Kinase dependencies associated with ovarian clear cell carcinoma. (Related to Figure 2). Boxplots illustrating the dependency of 11 ovarian clear cell (OCC) models on *CAMK2N1*, *GRK4* and *MAP3K9* relative to ovarian carcinoma models classified as other subtypes. See also Supplementary Table S1F. In each box plot the top and bottom of the box represents the third and first quartiles and the box band represents the median (second quartile); whiskers extend to 1.5 times the interquartile distance from the box.

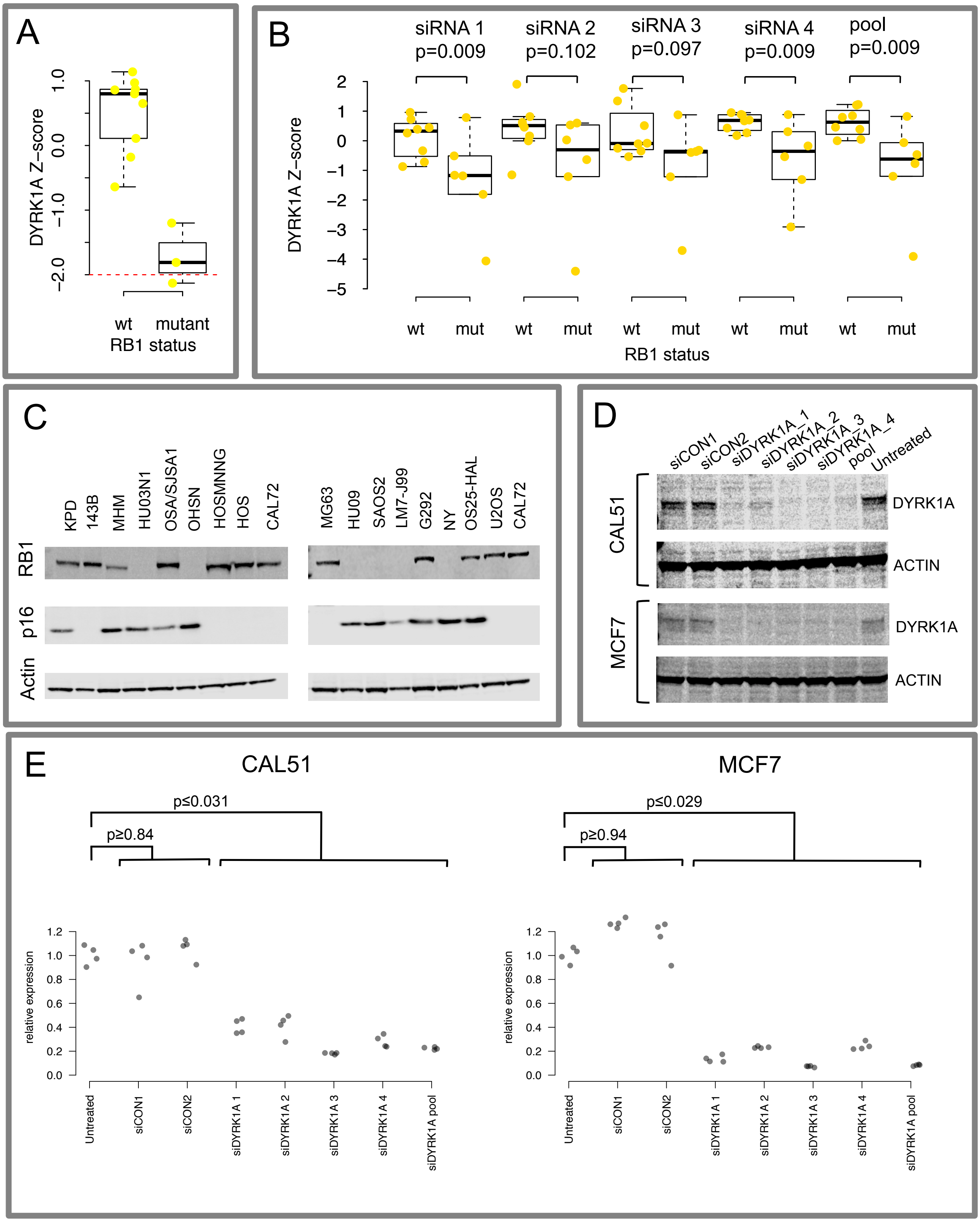

Figure S3. Validation of DYRK1A dependency in RB1 mutant osteosarcoma cell lines. (Related to Figure 3). A) Boxplot summarising Z-scores of osteosarcoma models treated with siRNAs targeting *DYRK1A* and grouped according to RB1 mutation status. B) *DYRK1A* siRNA smart pool deconvolution in *RB1* wt and mutant osteosarcoma models. P-values shown for each siRNA and the pool of siRNAs (siPool) are the result of applying median permutation tests to the siRNA Z-scores comparing the mutant and wild type groups. C) Detection of RB1, p16 (CDKN2A) and actin in osteosarcoma cell lines using western blotting and immunostaining of proteins. D) CAL51 and MCF7 cells treated with individual siRNAs targeting *DYRK1A* together with a pool of the same siRNAs, non-targeting negative control siRNAs (siCON1, siCON2) or untreated cells were harvested 72 h post transfection. DYRK1A and Actin protein abundance was evaluated using western blotting and immunostain detection. E) Expression of *DYRK1A vs GAPDH* mRNA in CAL51 and MCF7 cells treated with siRNAs. Relative expression values were normalized to the median of the untreated samples. Explanation as for D.

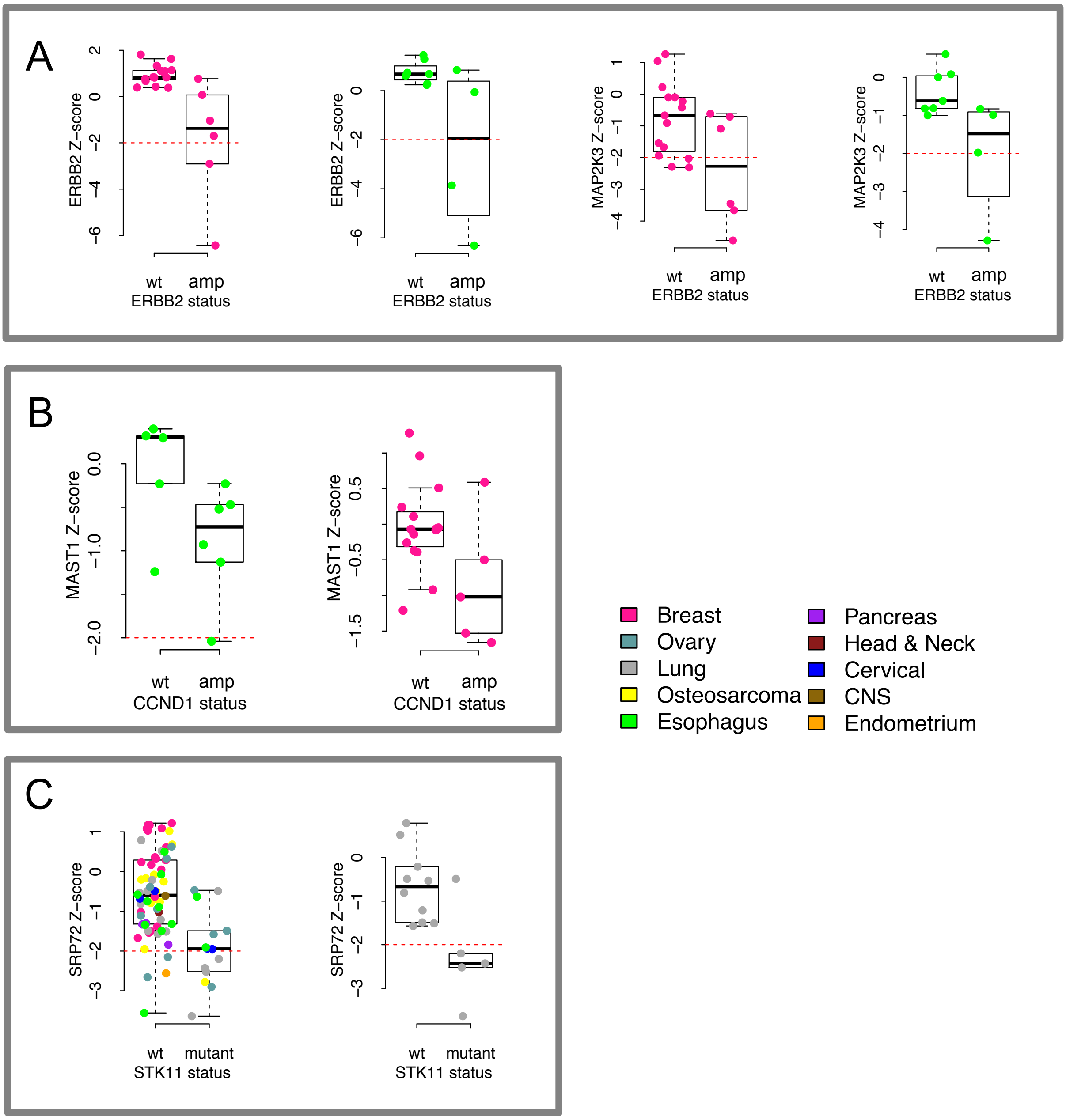

Figure S4. Boxplots showing kinase dependencies associated with driver gene mutations. (Related to Figure 3). A) Genetic dependencies associated with *ERBB2* amplification within breast and esophageal cancer histotypes. B) Genetic dependencies associated with *CCND1* amplification within breast and esophageal cancer histotypes. C) Dependency of *STK11* (*LKB1*) mutant cell lines to siRNA designed to target the Signal Recognition Particle 72 gene *SRP72* in the complete panel of cell lines and within the lung cancer panel. In each box plot the top and bottom of the box represents the third and first quartiles and the box band represents the median (second quartile); whiskers extend to 1.5 times the interquartile distance from the box.

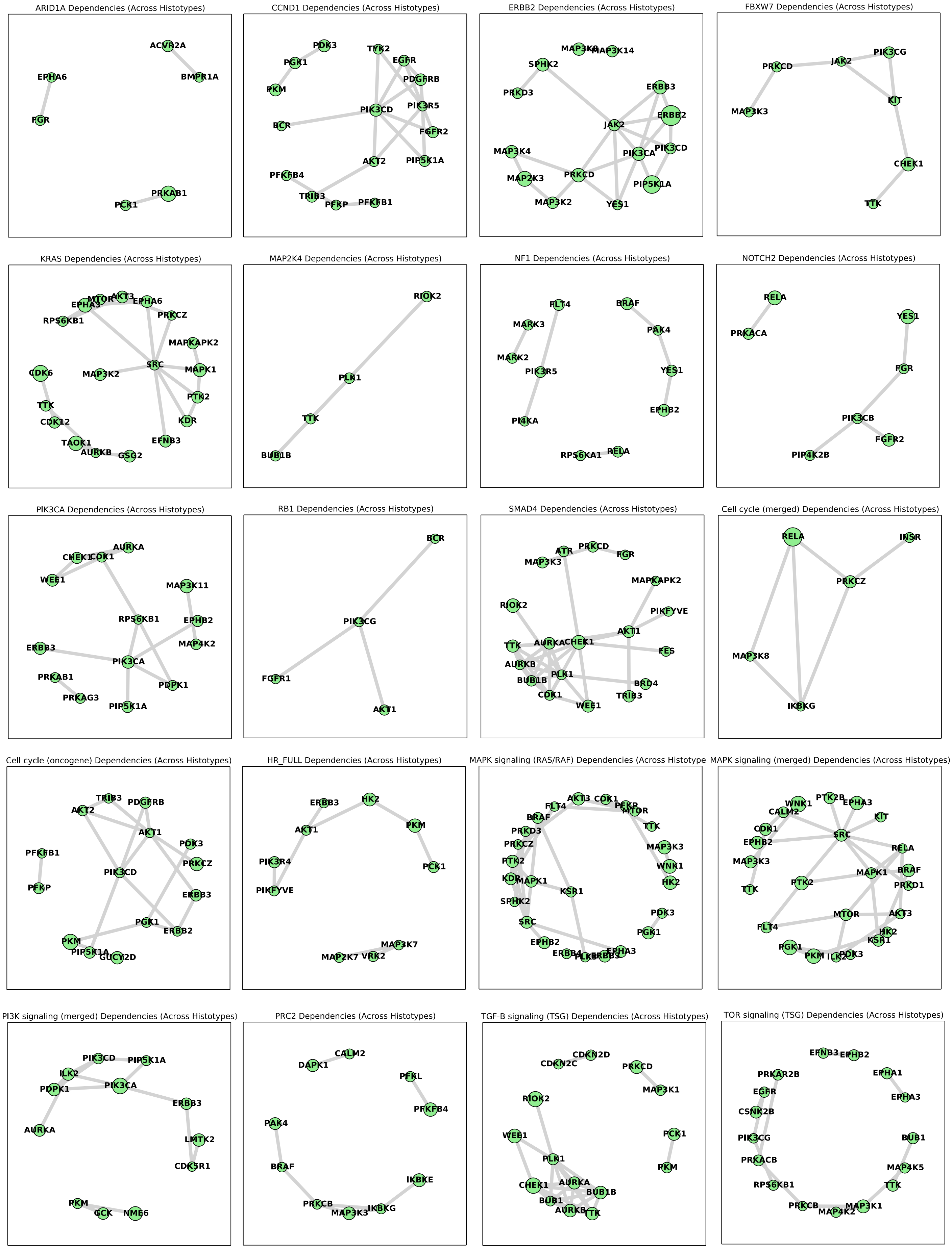

Figure S5. Network dependency maps for driver genes and pathways. (Related to Figure 4 and Figure 5). Functional interaction networks showing high-confidence STRING interactions between KGDs associated with 11 driver genes and 9 pathways. Only those networks that are more densely connected than expected are shown. See also Supplementary Table S1L

Table S1. Datasets used in the study and analysis result tables. The sheet named ‘explanation of the tables’ details the contents.

Supplemental Experimental Procedures

Cell lines and siRNA

The majority of tumor cell lines were obtained from the American Type Culture Collection, European Collection of Cell Cultures and Deutsche Sammlung von Mikroorganismen und Zellkulturen. Head and neck cancer cell line models were obtained from Susanne Gollin and Theresa Whiteside (University of Pittsburgh), Tom Carey (University of Michigan) and Hans Joenje (VU Medical Centre, NL). All cell lines were maintained as per the supplier's instructions. STR typing of 10 loci was performed on each cell line using the GenePrint 10 system (Promega) and used to confirm the identity of cell lines prior to storage. Cell lines were transfected with siRNA SMARTpools (Dharmacon) using Dharmafect 3, Dharmafect 4 (Dharmacon), Oligofectamine, Lipofectamine 2000 or RNAi max (Invitrogen) transfection reagents. siPLK1 (Dharmacon) was used as a positive control on each screen plate. Negative controls were siCON1, siCON2 (Dharmacon) and Allstar (Qiagen) and were included on every screening plate.

siRNA screening and processing

136 cancer-derived cell lines spanning 10 different tissue types were optimised for siRNA screening. This involved titrating the amount of lipid transfection reagent used as well as titrating cell number used for reverse siRNA transfections. For each cell line we tested at least four different transfection reagents and selected conditions that met the following criteria: (i) compared to a mock control (no lipid or siRNA), the transfection of non-silencing negative control siRNA caused no more than 20 % cell inhibition; (ii) compared to non-silencing negative control siRNA, the transfection of PLK1–targeting siRNA caused more than 80% cell inhibition; (iii) cell confluency reached 70% within the range of 4-7 days. The later criteria allowed assays to be terminated whilst cells were in growth phase. Once optimal conditions had been established, each cell line was transfected with a plate-arrayed siRNA library targeting 714 kinases and kinase-related genes (Dharmacon). 20 breast cancer cell lines were screened in a 96 well plate format, while the remainder were screened in a 384 well plate format (Supplementary Table S1A). Cell viability was estimated using a luminescent assay detecting cellular ATP levels (CellTitre-Glo, Promega). Luminescence values were processed using the cellHTS2 R package (Boutros et al., 2006). To evaluate the effect of each siRNA pool on cell viability, we log2 transformed the luminescence measurements and then centred these to the median value for each plate. The plate-centred data were scaled to the median absolute deviation (MAD) of the library as a whole to produce robust Z-scores (Figure 1A). All screens were performed in triplicate. Screens judged to have poor dynamic range (Z' factor ≤ 0) (Zhang et al., 1999) or poorly correlated replicates ($r \leq 0.7$) were excluded during an evaluation of screen quality. Subsequently, we retained kinase dependency profiles for 117 cell line models for further analysis.

Small molecule kinase inhibitor cell inhibition assays

Cell lines were profiled using two FGFR inhibitors (AZD4547 and PD173074) at eight different concentrations (0.5, 1, 5, 10, 50, 100, 500 and 1000 nM). A majority of the panel of cell lines profiled were derived from breast cancer and osteosarcomas with smaller numbers of cell lines derived from tumors of the central nervous system, cervix, head and neck, large intestine and lung cancer. 250-500 cells were seeded in 384 well plates. 24 hours post seeding, drug exposure was initiated and cells were continuously cultured in the presence of the drugs for a period of five days, at which point cell viability was estimated using Cell-Titre Glo (Promega). Cells were initially plated at a density to ensure that each cell line was in growth phase by the end of the five day treatment, similar to (Garnett et al., 2012). Luminescence values were normalised to the median of the per-plate DMSO negative control wells and the dose-response relationships modelled using 3-parameter logistic regression provided by the drc R-package (Ritz and Streibig, 2005). Area under the dose response curve (AUC) measurements calculated using this package were used as a read-out of drug sensitivity. Each cell line was assessed in triplicate.

Annotating cell lines according to mutation status

We annotated cell lines according to the presence of DNA mutations and copy number alterations in oncogenes and tumor suppressor genes. We included genes in the Cancer Gene Census (Futreal et al., 2004), genes listed in a census of amplified and overexpressed genes in human cancer (Santarius et al., 2010) and genes identified as candidate tumor suppressor gene (TSG) or oncogenes (OG) using the TUSON explorer approach described by (Davoli et al., 2013). The set of cancer genes were classified into

a tumor suppressor (TSG)-like group and an oncogene (OG)-like group using the molecular genetic classification information for genes available in the Cancer Gene Census. Genes with dominant effects were classified an oncogenes and those with recessive effects classified as tumor suppressor genes. Genes that were not included in the Cancer Gene Census were classified using the TUSON Explorer TSG and OG q-values (a positive classification occurring where the q-score for a given gene was < 0.1).

We obtained exome sequencing data for 77 cell lines from the COSMIC cell line project (http://cancer.sanger.ac.uk). We extended this data set with new exome sequencing data from 11 ovarian cancer cell lines (deposited at http://www.ebi.ac.uk/ena/data/view/PRJEB9639, exome pipeline described in a later section). We also obtained gene copy number data for 86 cell lines from the CCLE project (http://www.broadinstitute.org/ccle/) (Barretina et al., 2012). To annotate cell lines according to likely functionally mutated cancer genes we built a pipeline that takes mutation information from whole exome sequencing experiments and somatic copy number variation (CNVs) from multiple sources and classifies each cell line as to whether or not there is evidence for mutation or CNV for each of the cancer genes (https://github.com/GeneFunctionTeam/cell_line_functional_annotation). This annotation pipeline conforms to guidelines for the integration of functional genomics data proposed by the International Cancer Genome Consortium (Gonzalez-Perez et al., 2013). A dictionary of terms describing mutational consequences in the various data sets was used to enable comparison across each data set. Specifically, terms relating to copy number amplification, homozygous deletion, truncating mutations or recurrent missense mutations were used to define those mutations most likely to have a functional consequence in affected cell lines. Where a mutation was observed that was deemed to have an uncertain consequence (for example, a non-recurrent missense mutation), the event was recorded so that during the association tests any cell lines with such mutations in a gene could be excluded from both the mutant and wild-type group. The annotation pipeline also performed standardisation of cell line names and gene identifiers.

Using the exome data we considered a TSG-like gene to be functionally mutated in a given cell line if it contained a likely loss-of-function mutation (frameshift, nonsense or splice site alteration) or a missense mutation at a recurrently mutated residue (described in the next paragraph). In contrast we only considered an OG-like gene to be functionally mutated if it contained a mutation of a recurrently mutated residue. For CNV data sets we classified cell lines as containing a functionally relevant mutation if there was evidence of homozygous deletion of a TSG-like gene (GISTIC score of -2) or genomic amplification of an OG-like gene (GISTIC score of 2).

Even for well established cancer-associated genes, distinguishing between driver mutations (that have a likely gain of function effect for oncogenes or loss of function effect for tumor suppressors) and passenger mutations (that have no oncogenic effect) can be difficult for missense somatic mutations (point mutations). To address this problem we attempted to distinguish between the two by focussing on recurrent mutations - those mutations that alter residues that are frequently mutated across multiple tumor samples. We defined recurrently mutated residues using a database of > 1.2 million somatic mutations (Davoli et al., 2013). As some genes are sequenced more frequently than others (due to targeted sequencing) or mutated more frequently than others (due to genome location / chromatin accessibility) the threshold for defining a residue recurrently mutated is set on a per gene basis. For the analysis described here we defined a recurrently mutated residue as one that is mutated in either 3% of all samples featuring a mutation in that gene or three samples overall, whichever threshold is larger.

The set of functionally relevant alterations in cancer genes based on the exome and CNV data types were represented as Boolean matrices (1 for mutant, 0 for non-mutant). These datasets were combined using a simple logical OR function. For example, combining exome and CNV data sets we considered an oncogene to be mutated in a given cell line if the oncogene was either amplified according to the CNV data OR mutated at a recurrently altered site according to the exome data. Cell lines where a given data type was not available were excluded from analyses incorporating that data type.

Exome sequencing pipeline

Exome sequencing libraries were prepared using SureSelect Human All Exon 50 Mbp kits (Agilent). Illumina paired-end libraries were sequenced on a HiSeq2000 (Illumina), acquiring 2 x 76 bp reads. Basecalling and demultiplexing was performed using Casava v1.8 (Illumina) software. Fastq files were

aligned to the human reference genome (GRCh37) using the Burrows-Wheeler Aligner (Li and Durbin, 2009). Duplicate reads arising from PCR were removed prior to further processing and variant detection. Base recalibration, realignment around indels and variant calling were performed using the Genome Analysis Tool-kit v2 with default settings (DePristo et al., 2011). Variants called in regions not covered by the capture probes were excluded, as were those with genotype quality scores below 20 and those covered by fewer than 10 reads.

Protein Quantitation
Whole cell protein lysates were extracted from cells by lysis with NP250 buffer (20 mM Tris pH 7.6, 1 mM EDTA, 0.5%. NP40, 250 mM NaCl). For RNAi knock-down, samples were extracted 72 hours post transfection. Western blots were performed using Novex precast TA gels (Invitrogen) as described previously (Farmer et al, 2005). Primary antibodies were immunoblotted overnight using either anti-RB1 (1/1000; Cell Signaling), anti-DYRK1A (1/1000 Cell Signalling), anti-p16 (1/1000 Abcam) or anti-ACTIN (1/2000; Santa Cruz). Fluorescent anti-rabbit, anti-mouse or anti-goat secondary antibodies (Licor) were incubated with the blot (1:5000) for 1 hour at room temperature in the dark followed by detection and processing on a Licor Odyssey Western Imager. Blots were viewed using ImageStudio Software (Licor).

Quantitative RT–PCR
Total RNA from cell lines were extracted using RNeasy mini kit (Qiagen) according to the manufacturer's instructions. cDNA was synthesised using SuperScript III reverse transcriptase (Invitrogen) for RT–PCR with random hexamers as per the manufacturer's instructions. Assay-on-Demand primer/probe sets were purchased from Applied Biosystems. Real-Time qPCR was performed on the 7900 DHT Fast Real-Time PCR System (Applied Biosystems), using GAPDH as an endogenous control. Gene expression was calculated relative to expression of GAPDH endogenous control. Samples were quantified in quadruplicate.

Data Integration
Protein-protein interactions were obtained from the HINT (Das and Yu, 2012), BioGRID version 3.4.128 (Chatr-Aryamontri et al., 2015) and KEA databases (Lachmann and Ma'ayan, 2009). Kinase-substrate interactions were obtained from KEA (Lachmann and Ma'ayan, 2009), PhosphoSitePlus (Hornbeck et al., 2015) and (Cheng et al., 2014). High confidence (combined score > 0.7) functional interactions were obtained from the STRING database (Version 9.1; (Franceschini et al., 2013)). Gene expression relationships were obtained from PathwayCommons (Cerami et al., 2011) by extracting those relationships annotated as 'controls-expression-of'. To identify the shortest directed path between driver genes and their dependencies we built an integrated directed network using the directed edges from PathwayCommons (those labelled 'controls-expression-of', 'controls-phosphorylation-of', 'controls-transport-of', 'controls-production-of') and the full set of kinase-substrate interactions from all sources. The shortest_path function in NetworkX (Hagberg et al., 2008) was then used to query this graph. All shortest paths of length 2 or less are provided in Supplemental Table S1I and S1K. To evaluate the connectivity of the dependencies associated with each driver gene we compared the number of observed high-confidence STRING interactions between the nominally significant dependencies (those with median permutation test p-value < 0.05) to the number observed on 100 randomized degree matched interaction networks.

Pathway analysis
The original pathway groupings were taken from (Garraway and Lander, 2013). Four groupings were dropped because they only contain a single gene (e.g. the 'RNA abundance' pathway contained a single gene *DIS3*). Six groupings were removed because they referred to a broad molecular class of genes ('RTK Signalling', 'Chromatin histone methyltransferases') rather than a specific pathway or complex. Three further groupings were removed as they were catch-all terms for unclassified driver genes (e.g. 'Other signaling'). We then added three additional pathways to this list – homologous recombination (based on data described by Wood and Lindahl (Wood et al., 2005; Wood et al., 2001), FGFR signalling (containing the FGFR genes which were previously only annotated as 'RTK signaling') and the PRC2 complex (Kuzmichev et al., 2002). From this list we then removed groupings that were not represented by any mutant cell lines in our panel or groupings in which only a single member of the grouping was mutated in our cell lines. Finally pathways that were essentially redundant (comprised of identical sets of

mutated genes) in the panel of cell lines we screened were combined. The pathways used are presented in Table S1M.

Supplemental references

Barretina, J., Caponigro, G., Stransky, N., Venkatesan, K., Margolin, A.A., Kim, S., Wilson, C.J., Lehar, J., Kryukov, G.V., Sonkin, D., *et al.* (2012). The Cancer Cell Line Encyclopedia enables predictive modelling of anticancer drug sensitivity. Nature *483*, 603-607.

Boutros, M., Bras, L.P., and Huber, W. (2006). Analysis of cell-based RNAi screens. Genome Biol *7*, R66.

Cerami, E.G., Gross, B.E., Demir, E., Rodchenkov, I., Babur, O., Anwar, N., Schultz, N., Bader, G.D., and Sander, C. (2011). Pathway Commons, a web resource for biological pathway data. Nucleic Acids Res *39*, D685-690.

Chatr-Aryamontri, A., Breitkreutz, B.J., Oughtred, R., Boucher, L., Heinicke, S., Chen, D., Stark, C., Breitkreutz, A., Kolas, N., O'Donnell, L., *et al.* (2015). The BioGRID interaction database: 2015 update. Nucleic Acids Res *43*, D470-478.

Cheng, F., Jia, P., Wang, Q., and Zhao, Z. (2014). Quantitative network mapping of the human kinome interactome reveals new clues for rational kinase inhibitor discovery and individualized cancer therapy. Oncotarget *5*, 3697-3710.

Das, J., and Yu, H. (2012). HINT: High-quality protein interactomes and their applications in understanding human disease. BMC Syst Biol *6*, 92.

Davoli, T., Xu, A.W., Mengwasser, K.E., Sack, L.M., Yoon, J.C., Park, P.J., and Elledge, S.J. (2013). Cumulative haploinsufficiency and triplosensitivity drive aneuploidy patterns and shape the cancer genome. Cell *155*, 948-962.

DePristo, M.A., Banks, E., Poplin, R., Garimella, K.V., Maguire, J.R., Hartl, C., Philippakis, A.A., del Angel, G., Rivas, M.A., Hanna, M., *et al.* (2011). A framework for variation discovery and genotyping using next-generation DNA sequencing data. Nat Genet *43*, 491-498.

Franceschini, A., Szklarczyk, D., Frankild, S., Kuhn, M., Simonovic, M., Roth, A., Lin, J., Minguez, P., Bork, P., von Mering, C., *et al.* (2013). STRING v9.1: protein-protein interaction networks, with increased coverage and integration. Nucleic Acids Res *41*, D808-815.

Futreal, P.A., Coin, L., Marshall, M., Down, T., Hubbard, T., Wooster, R., Rahman, N., and Stratton, M.R. (2004). A census of human cancer genes. Nature Rev Cancer *4*, 177-183.

Garraway, L.A., and Lander, E.S. (2013). Lessons from the cancer genome. Cell *153*, 17-37.

Gonzalez-Perez, A., Mustonen, V., Reva, B., Ritchie, G.R., Creixell, P., Karchin, R., Vazquez, M., Fink, J.L., Kassahn, K.S., Pearson, J.V., *et al.* (2013). Computational approaches to identify functional genetic variants in cancer genomes. Nat Methods *10*, 723-729.

Hagberg, A., Schult, D., and Swart, P. (2008). Exploring Network Structure, Dynamics, and Function using NetworkX. Proceedings of the 7th Python in Science Conference.

Hornbeck, P.V., Zhang, B., Murray, B., Kornhauser, J.M., Latham, V., and Skrzypek, E. (2015). PhosphoSitePlus, 2014: mutations, PTMs and recalibrations. Nucleic Acids Res *43*, D512-520.

Kuzmichev, A., Nishioka, K., Erdjument-Bromage, H., Tempst, P., and Reinberg, D. (2002). Histone methyltransferase activity associated with a human multiprotein complex containing the Enhancer of Zeste protein. Genes Dev *16*, 2893-2905.

Lachmann, A., and Ma'ayan, A. (2009). KEA: kinase enrichment analysis. Bioinformatics *25*, 684-686.

Li, H., and Durbin, R. (2009). Fast and accurate short read alignment with Burrows-Wheeler transform. Bioinformatics *25*, 1754-1760.

Ritz, C., and Streibig, J.C. (2005). Bioassay Analysis using R. J Statist Software *12*, 1-22.

Santarius, T., Shipley, J., Brewer, D., Stratton, M.R., and Cooper, C.S. (2010). A census of amplified and overexpressed human cancer genes. Nature Rev Cancer *10*, 59-64.

Wood, R.D., Mitchell, M., and Lindahl, T. (2005). Human DNA repair genes, 2005. Mutat Res *577*, 275-283.

Wood, R.D., Mitchell, M., Sgouros, J., and Lindahl, T. (2001). Human DNA repair genes. Science *291*, 1284-1289.

Zhang, J.H., Chung, T.D., and Oldenburg, K.R. (1999). A Simple Statistical Parameter for Use in Evaluation and Validation of High Throughput Screening Assays. J Biomol Screen *4*, 67-73.
