# Supplementary material for: Large-Scale Profiling of Kinase Dependencies in Cancer Cell Lines
Source: Cell Rep. 2016 Mar 3;14(10):2490–501. doi: 10.1016/j.celrep.2016.02.023 (PMC4802229; doi:10.1016/j.celrep.2016.02.023)
Supplement: Document S2. Article plus Supplemental Information [file mmc3.pdf]

Cell Reports

Article

# Large-Scale Profiling of Kinase Dependencies in Cancer Cell Lines

## Graphical Abstract

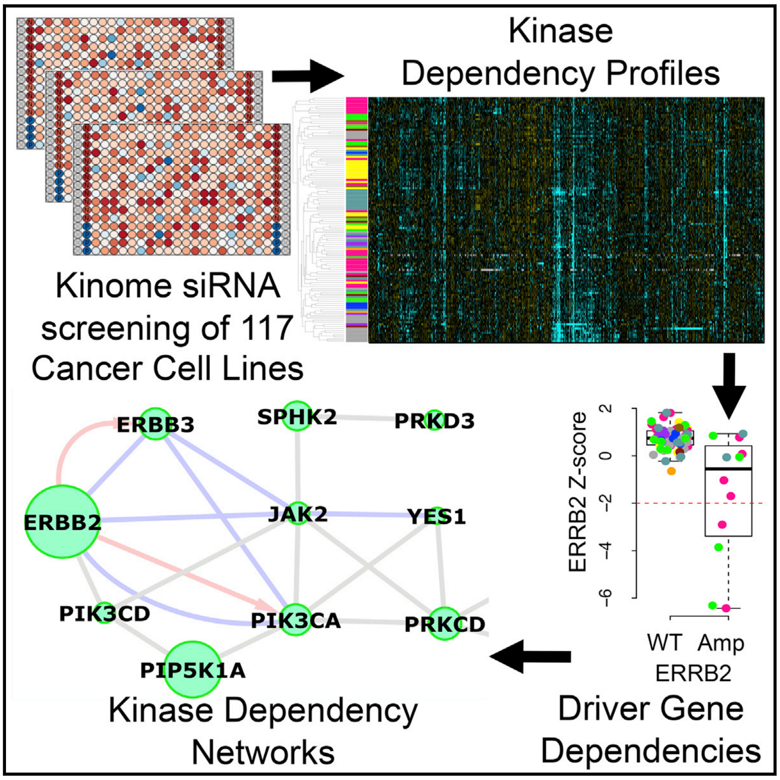


## Highlights

- Kinome-wide (714 gene) siRNA screens in 117 cell lines from ten cancer histotypes
- Integrating genotype data reveals cancer driver gene dependencies
- Integrating protein interaction data aids the interpretation of genetic dependencies
- Identified dependencies enable prediction of mutant cell line responses to drugs

## Authors

James Campbell, Colm J. Ryan, Rachel Brough, ..., Sandra J. Strauss, Alan Ashworth, Christopher J. Lord

## Correspondence

alan.ashworth@ucsf.edu (A.A.),
chris.lord@icr.ac.uk (C.J.L.)

## In Brief

Campbell et al. use parallel siRNA screens to identify the kinase dependencies of 117 cancer cell lines from ten cancer types. They use this resource to identify kinase dependencies associated with specific cancer types or driver genes and show that the integration of protein interaction networks facilitates the interpretation of these dependencies.

Campbell et al., 2016, Cell Reports *14*, 2490–2501
March 15, 2016 ©2016 The Authors
http://dx.doi.org/10.1016/j.celrep.2016.02.023

OPEN ACCESS
CellPress

Cell Reports
Article

# Large-Scale Profiling of Kinase Dependencies in Cancer Cell Lines

James Campbell,[1,9] Colm J. Ryan,[2,9] Rachel Brough,[1] Ilirjana Bajrami,[1] Helen N. Pemberton,[1] Irene Y. Chong,[1,3] Sara Costa-Cabral,[1] Jessica Frankum,[1] Aditi Gulati,[1] Harriet Holme,[1,4] Rowan Miller,[1,4] Sophie Postel-Vinay,[1,5] Rumana Rafiq,[1] Wenbin Wei,[1] Chris T. Williamson,[1] David A. Quigley,[6] Joe Tym,[7] Bissan Al-Lazikani,[7] Timothy Fenton,[4] Rachael Natrajan,[8] Sandra J. Strauss,[4] Alan Ashworth,[1,10,*] and Christopher J. Lord[1,*]

[1]The Breast Cancer Now Research Centre and CRUK Gene Function Laboratory, The Institute of Cancer Research, London SW3 6JB, UK
[2]Systems Biology Ireland, University College Dublin, Dublin 4, Ireland
[3]Royal Marsden Hospital, London SW3 6JJ, UK
[4]UCL Cancer Institute, University College London, London WC1E 6DD, UK
[5]Gustave Roussy Cancer Campus, 94805 Villejuif, France
[6]UCSF Helen Diller Family Comprehensive Cancer Centre, San Francisco, CA 94158, USA
[7]Cancer Research UK Cancer Therapeutics Unit, The Institute of Cancer Research, Sutton SM2 5NG, UK
[8]Functional Genomics Laboratory, The Breast Cancer Now Research Centre, The Institute of Cancer Research, London SW3 6JB, UK
[9]Co-first author
[10]Present address: UCSF Helen Diller Family Comprehensive Cancer Centre, San Francisco, CA 94158, USA
*Correspondence: alan.ashworth@ucsf.edu (A.A.), chris.lord@icr.ac.uk (C.J.L.)
http://dx.doi.org/10.1016/j.celrep.2016.02.023
This is an open access article under the CC BY license (http://creativecommons.org/licenses/by/4.0/).

## SUMMARY

**One approach to identifying cancer-specific vulnerabilities and therapeutic targets is to profile genetic dependencies in cancer cell lines. Here, we describe data from a series of siRNA screens that identify the kinase genetic dependencies in 117 cancer cell lines from ten cancer types. By integrating the siRNA screen data with molecular profiling data, including exome sequencing data, we show how vulnerabilities/genetic dependencies that are associated with mutations in specific cancer driver genes can be identified. By integrating additional data sets into this analysis, including protein-protein interaction data, we also demonstrate that the genetic dependencies associated with many cancer driver genes form dense connections on functional interaction networks. We demonstrate the utility of this resource by using it to predict the drug sensitivity of genetically or histologically defined subsets of tumor cell lines, including an increased sensitivity of osteosarcoma cell lines to FGFR inhibitors and SMAD4 mutant tumor cells to mitotic inhibitors.**

## INTRODUCTION

The phenotypic and genetic changes that occur during tumorigenesis alter the set of genes upon which cells are dependent. The best known example of this phenomenon of "genetic dependency" is oncogene addiction where tumor cells become dependent upon the activity of a single oncogene, which when inhibited leads to cancer cell death. Alternatively, tumor cells can become addicted to the activity of genes other than oncogenes, effects known as non-oncogene addictions (Luo et al., 2009), induced essential effects (Tischler et al., 2008), or synthetic lethal interactions (Kaelin, 2005). From a clinical perspective, identifying genetic dependencies in tumor cells could illuminate vulnerabilities that might be translated into therapeutic approaches to treat the disease. Examples of this approach include the development of drugs that target oncogene addiction effects, such as imatinib in the case of *ABL* addiction, and therapeutic approaches that exploit synthetic lethal effects, such as PARP inhibitors for *BRCA*-deficient cancers (Lord et al., 2015).

A number of groups have used high-throughput screening approaches such as RNAi or small molecule sensitivity screens to systematically identify genetic dependencies in tumor cell lines (Barretina et al., 2012; Brough et al., 2011; Cowley et al., 2014; Garnett et al., 2012; Koh et al., 2012). A particular focus has been in dissecting genetic dependencies that involve kinases (Brough et al., 2011; Grueneberg et al., 2008), as these enzymes play key roles in a number of oncogenic processes (Greenman et al., 2007) and are pharmacologically tractable (Sakharkar and Sakharkar, 2007; Workman and Al-Lazikani, 2013; Zhang et al., 2009). Previously, we used high-throughput short interfering (si)RNA screening to identify the kinase dependencies in a panel of 20 breast cancer derived cell lines (Brough et al., 2011). Here, we describe as a resource an expansion of this approach, namely parallel siRNA screens targeting 714 genes in 117 genetically and histologically diverse tumor cell lines. Building on our previous work (Brough et al., 2011), we extend our analytical approach to describe how this data set may be used as a hypothesis-generating tool for identifying candidate therapeutic targets associated with specific tumor histotypes or mutations in cancer driver genes. We also illustrate how, by integrating this functional data with orthogonal data sources such as protein-protein interaction data sets, these genetic dependencies might be dissected mechanistically.

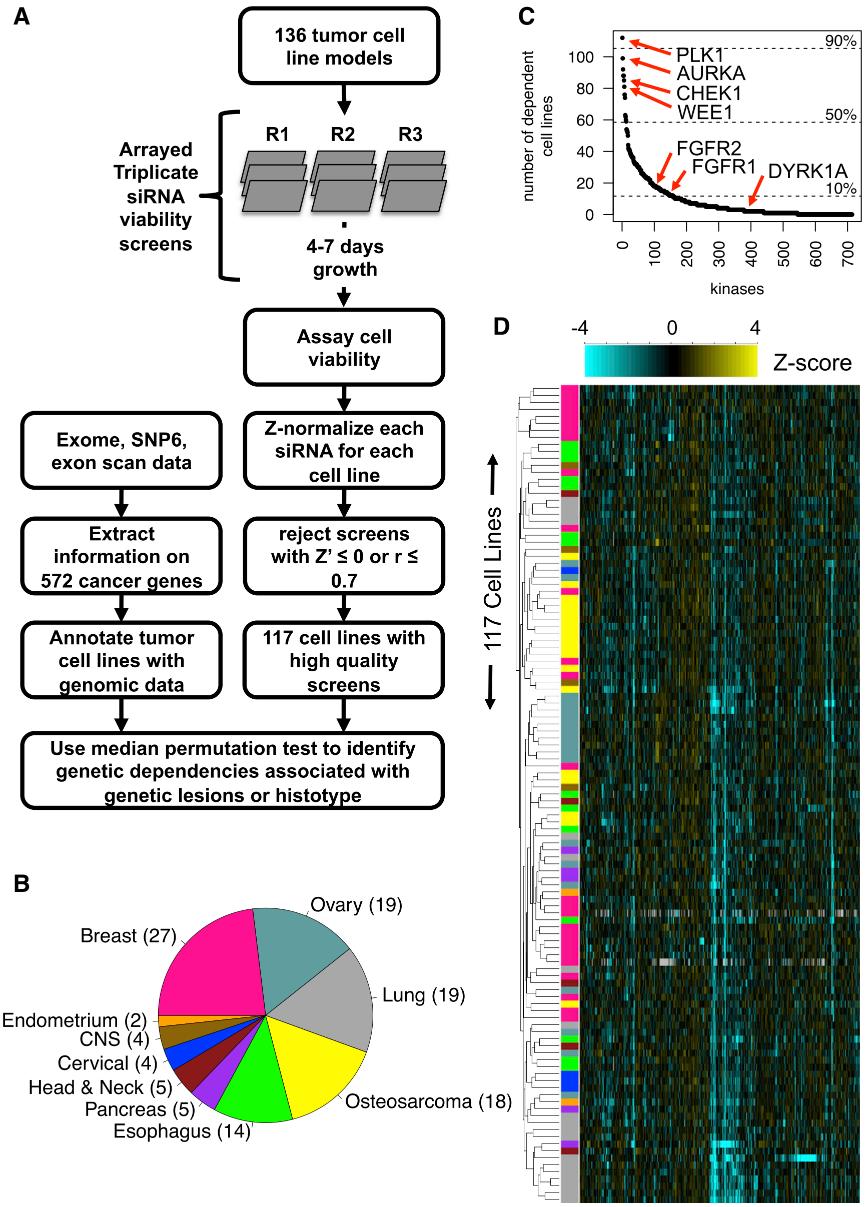


**Figure 1. Screening Overview**

(A) Schematic of siRNA screening, data processing, and genomic data integration.

(B) Piechart illustrating histotypes for 117 cell lines that passed QC (CNS).

(C) Frequency plot depicting the number of cell lines in which each kinase siRNA caused a significant growth defect ($Z \leq -2$).

(D) Clustered heatmap summarizing the KGDs of 117 cell lines. The average linkage hierarchical clustering was used with Pearson's correlation as the similarity metric. Only the 20% most variable siRNA $Z$ scores were used for the calculation of correlations. The histotype of each cell line is indicated by the color blocks to the left of the heatmap and corresponds to the scheme shown in (B).

## RESULTS

### Kinase Genetic Dependencies Identified by Parallel siRNA Screens

We screened a panel of 136 tumor cell lines in triplicate in a plate-arrayed format using an siRNA library designed to target 714 genes (see Experimental Procedures and Figure 1A). The genes targeted with this library included 500 protein kinases (Manning et al., 2002), with the remaining targets comprising metabolic kinases (e.g., ATP-dependent 6-phosphofructokinases), lipid kinases (e.g., PIK3C2A), as well as proteins that lack kinase activity, but directly impact kinase signaling (e.g., the cyclin dependent kinase inhibitor CDKN1C). Cells were reverse transfected with siRNA and then cultured until cells reached 70% confluency (on average 4-7 days), at which point cell viability was assessed using the CellTiter-Glo assay. Following data processing and the application of quality control filters to ensure the reproducibility and high dynamic range of each screen (see Supplemental Information; Figure 1A; Table S1A), we retained 117 high quality screens for further analysis. The resulting resource (Table S1B) features tumor cell line models from ten different cancer types (breast, ovarian, lung, osteosarcoma, esophageal, pancreatic, head, and neck, cervical, CNS, and endometrioid; Figure 1B), and includes data for 69 lines not profiled in prior large-scale RNAi screens (Brough et al., 2011; Cowley et al., 2014; Koh et al., 2012).

To allow data to be compared between different cell lines, the viability data from each screen were standardized by the use of a robust $Z$ score statistic (Table S1B). We considered candidate kinase genetic dependencies (KGDs) in the data set as those where the siRNA elicited $Z < -2$ effects. 76% of the kinases profiled in the screening library represented KGDs in at least one tumor cell model. Additionally, 53% and 26% represented KGDs in $\geq$5 and $\geq$10 cell lines, respectively (Figure 1C and Table S1C). On average, each tumor cell line model exhibited 51 KGDs. A set of six kinase-coding genes (*PLK1*, *AURKA*, *WEE1*, *CHEK1, CDK11A*, and *GUCY2D*) represented KGDs in >70% of the cell lines screened and four of these (*PLK1*, *AURKA*, *WEE1*, and *CHEK1*) are known to be involved in the mitotic cell-cycle -checkpoint.

### Candidate KGDs Associated with Tumor Histotypes

Using average linkage hierarchical clustering to cluster the siRNA $Z$ score data (Figure 1D), we found that tumor cell lines frequently clustered according to tumor histotype. For example,

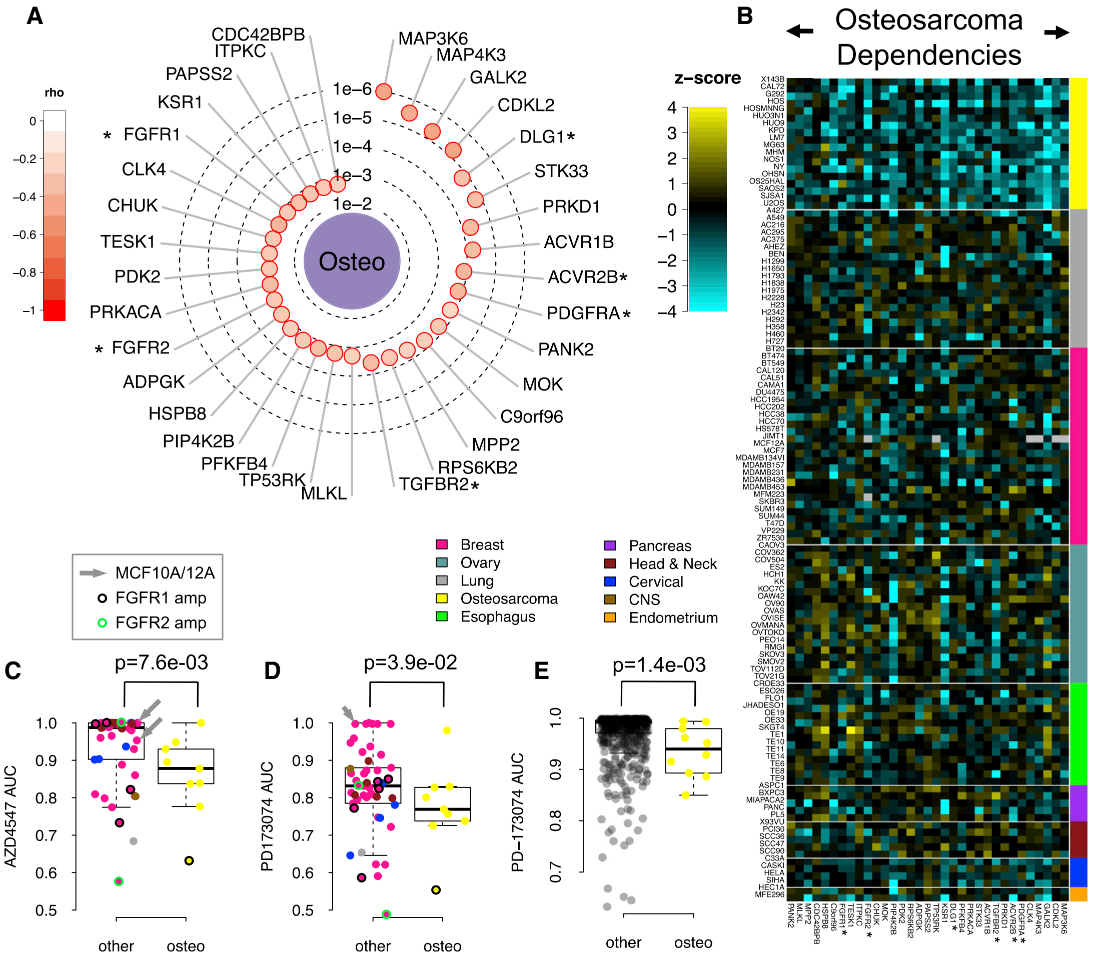


**Figure 2. Kinase Dependencies Associated with Histotypes**

(A) Radar plot summarizing the KGDs associated with the osteosarcoma histotype. The concentric circles indicate the statistical significance and the depth of color indicates the separation of *Z* scores between the osteosarcoma histotype and the non-osteosarcoma group of cell lines. A set of six kinases annotated as involved in skeletal system morphogenesis in the Gene Ontology are annotated with asterisks.

(B) Heatmap of KGDs enriched in osteosarcoma cell lines are shown as a heatmap representing siRNA *Z* scores. The asterisks indicate kinases involved in skeletal system morphogenesis as in (A).

(C and D) Box plots of area under curve (AUC) estimates for 58 cell lines exposed to the FGFR inhibitor AZD4547 (C) and PD173074 (D) at eight different concentrations. *FGFR1* and *FGFR2*-amplified cell lines are indicated with black and green circles, respectively. The non-tumor epithelial cell lines MCF10A and MCF12A are indicated with gray arrows.

(E) Box plot of AUC estimates for a panel of cell lines exposed to the FGFR inhibitor PD173074 (Garnett et al., 2012).

In each box plot (C–E), the top and bottom of the box represents the third and first quartiles and the box band represents the median (second quartile); whiskers extend to 1.5 times the interquartile distance from the box. See also Figures S1 and S2.

the majority of ovarian cancer cell lines formed a single cluster, as did those models derived from osteosarcomas (Figure 1D). Using median permutation (MP) tests on the *Z* score data, we found 151 KGDs associated with specific histotypes at a false discovery rate (FDR) of 0.1 (Table S1D). As expected, the number of KGDs associated with each histotype was correlated with the number of cell lines screened for that histotype (Spearman's rho = 0.82), reflecting the increased statistical power resulting from a larger sample size. In breast cancer models, we found an increased requirement for *ERBB3* and *PIK3CA*, members of the ERBB2 and PI3-kinase signaling pathways that are frequently dysregulated in this cancer histology (Miller et al., 2011). In contrast, models of osteosarcoma were more reliant upon genes involved in "skeletal system morphogenesis", including *PDGFRA*, *ACVR2B*, *TGFBR2*, *DLG1*, *FGFR1*, and *FGFR2* (Su et al., 2008) (Gene Ontology enrichment $p < 0.001$ after correcting for multiple hypothesis testing, Berriz et al., 2009; Figures 2A and 2B). The *FGFR1* and *FGFR2* KGDs suggested that osteosarcoma models might be especially sensitive to small molecule FGFR inhibitors. Testing a set of 58 tumor cell lines for FGFR inhibitor sensitivity, we found AZD4547 (Gavine et al., 2012) and PD173074 (Bansal et al., 2003) to be more selective for osteosarcoma models (AZD4547, $p = 7.6 \times 10^{-3}$, PD173074 $p = 3.9 \times 10^{-2}$; Figures 2C and 2D; Table S1E) and

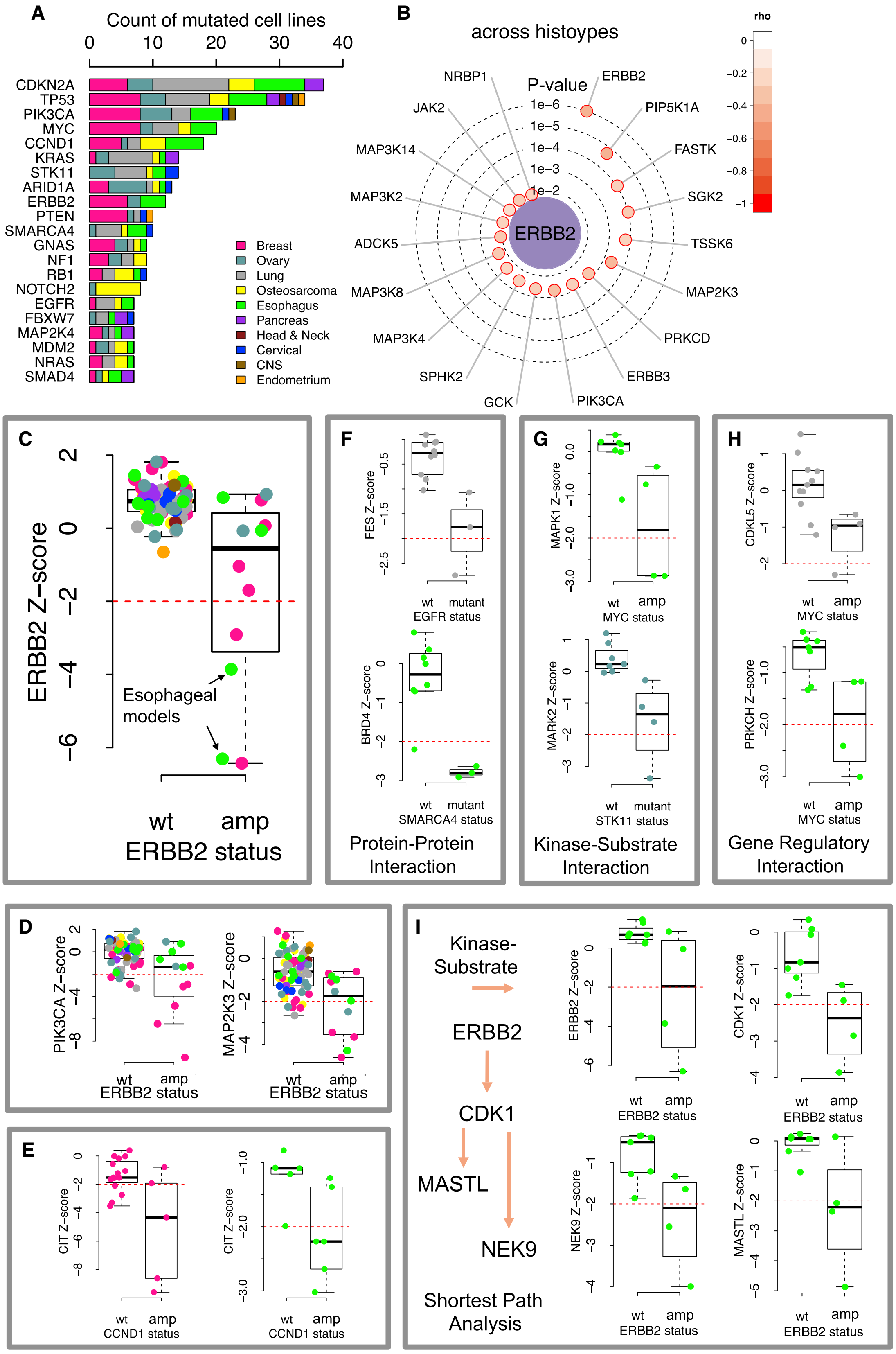


(legend on next page)

to have minimal effects in two non-tumor epithelial models (Figure S1). This osteosarcoma selective effect was independent of *FGFR1* or *FGFR2* amplification status and was also apparent when *FGFR1* or *FGFR2* amplified tumor cell lines were excluded from the analysis (AZD4572, $p = 7.2 \times 10^{-3}$ and PD173074, $p = 4.3 \times 10^{-2}$; Figures 2C and 2D). Furthermore, the osteosarcoma selective nature of PD173074 was confirmed by a reanalysis of PD173074 sensitivity data derived from 660 tumor cell lines (Garnett et al., 2012) (Figure 2E; $p = 1.4 \times 10^{-3}$). Taken together, these results suggested that FGFR inhibitors might show some utility in osteosarcoma, but that factors in addition to *FGFR1* and *FGFR2* amplification might explain drug sensitivity in this setting.

We also assessed the possibility that KGDs could be identified that were associated with specific subtypes of cancer. We, and others, have previously used RNAi data to identify KGDs associated with distinct breast cancer subtypes (Brough et al., 2011; Marcotte et al., 2012). To illustrate the utility of the expanded data set described here, we used MP tests to identify KGDs associated with the clear cell subtype of ovarian cancer (OCC). We found three kinases (*CAMK2N1*, *GRK2*, and *MAP3K9*) to be KGDs in OCC models, compared to other ovarian cancer histologies such as serous ovarian cancer (Figure S2; Table S1F).

## Candidate KGDs Associated with Driver Gene Alterations

By integrating the siRNA data with exome sequencing (Forbes et al., 2015) and copy number profiling data (Barretina et al., 2012), we identified KGDs associated with mutations in each of 200 candidate cancer driver genes (see Supplemental Information; Table S1G). We identified 4,247 putative dependencies associated with driver gene mutations (uncorrected MP test $p \leq 0.05$; Table S1H). As the large number of tests performed using these 200 driver genes prohibited correction for multiple hypothesis testing, we focused our subsequent analysis on 21 key cancer driver genes (12 tumor suppressor genes and nine oncogenes; Futreal et al., 2004; Vogelstein et al., 2013) (Figure 3A) mutated in at least seven tumor cell lines in our panel. This identified 211 KGDs at an FDR of 0.5 (Table S1I) that could form the basis for subsequent validation experiments.

This approach reconfirmed the well-established *ERBB2* oncogene addiction in models of breast cancer, but also established *ERBB2* addiction/KGD in models of esophageal cancer (Figures 3B and 3C), where *ERBB2* is recurrently amplified/overexpressed in 20% of tumors (Bang et al., 2010). This suggested that this particular genetic dependency was relatively independent of the underlying histotype. *ERBB2* amplification was also associated with dependency upon other members of the ERBB2 signaling network including the ERBB2 binding partner ERBB3 ($p = 2 \times 10^{-3}$), JAK2 ($p = 1 \times 10^{-2}$), and the downstream effector of ERBB2, PIK3CA ($p = 4 \times 10^{-3}$; Figure 3D). We found other KGDs associated with *ERBB2* amplification, including a strong dependency upon the stress response kinase MEK3 (*MAP2K3,* $p = 4 \times 10^{-4}$; Figure 3D) (Dérijard et al., 1995) and *PIP5K1A* ($p = 2 \times 10^{-5}$), a kinase involved in inositol phosphate metabolism (Loijens and Anderson, 1996).

We assessed the possibility that some KGDs associated with cancer driver gene mutations might be private to or more profound in particular histotypes. For example, *BRAF* p.V600E mutant melanomas are extremely sensitive to BRAF inhibition, whereas colorectal cancers with the same mutation show little response (Prahallad et al., 2012). We used a similar analysis as above to identify KGDs associated with driver gene mutations within particular histotypes and identified 943 KGDs (Tables S1J and S1K), compared to 211 in the prior analysis that combined all histotypes. Together, these 1,154 candidate dependencies could inform the design of subsequent validation studies. As an example of this, we selected for validation one of the KGDs associated with *RB1* mutation in osteosarcoma (Kansara et al., 2014), DYRK1A ($p = 6.8 \times 10^{-3}$; Figure S3A), a component of the DREAM complex (Sadasivam and DeCaprio, 2013) previously identified as a protein interaction partner of RB1 (Varjosalo et al., 2013). To confirm the dependency of *RB1* null osteosarcoma models upon *DYRK1A*, we selected 14 osteosarcoma models and characterized these according to their *RB1* mutation and protein expression status and established that multiple distinct *DYRK1A* siRNAs could replicate the *RB1* selectivity observed in the initial screen as well as eliciting *DYRK1A* silencing (Figure S3). These results suggest that *DYRK1A* might represent a valid genetic dependency in *RB1* defective osteosarcoma cells.

We also noted from our analysis of the siRNA data that some genetic dependencies associated with cancer driver gene mutations were observed independently in multiple histotypes. These included KGDs associated with *ERBB2* amplification in breast and esophageal cancer models (e.g., *ERBB2* $p = 7.9 \times 10^{-5}$ [breast] and $p = 9.2 \times 10^{-3}$ [esophageal] and *MAP2K3* $p = 3.3 \times 10^{-2}$ [breast] and $p = 4.4 \times 10^{-3}$ [esophageal]; Figure S4A), but also a dependency upon the microtubule associated

**Figure 3. KGDs Associated with Cancer Driver Mutations**

(A) Bar chart indicating the frequency of driver gene alterations observed in the cell line panel. The colored segments in each bar indicate the histotypes in which alterations were detected.

(B) Radar plot summarizing the KGDs associated with *ERBB2* amplification (the scheme as per Figure 2A).

(C) Box plot showing the ERBB2 *Z* scores for cell lines grouped according to *ERBB2* amplification status. The colors indicate cell line histotypes as in (A).

(D) Box plots showing additional KGDs associated with *ERBB2* amplification.

(E) Box plots summarizing *CCND1* KGDs upon *CIT*.

(F) Examples of KGDs that are supported by protein-protein interactions.

(G) Examples of KGDs that are supported by kinase-substrate relationships.

(H) Examples of KGDs that are supported by gene regulatory relationships.

(I) Examples of KGDs associated with *ERBB2* amplification status in esophageal cancer models supported by kinase-substrate relationships that form a shortest path between the mutated driver gene and kinases.

In each box plot (C–I), the top and bottom of the box represents the third and first quartiles and the box band represents the median (second quartile); whiskers extend to 1.5 times the interquartile distance from the box. See also Figures S3 and S4 and Tables S1I and S1K.

serine/threonine kinase *MAST1* in *CCND1* amplified breast or esophageal cancer models ($p = 1.1 \times 10^{-2}$ [breast] and $p = 1.3 \times 10^{-2}$ [esophageal]; Figure S3B). Likewise, a KGD upon Citron Rho-interacting kinase *(CIT)*, a regulator of cytokinesis (Madaule et al., 1998) was also seen in *CCND1* amplified breast or esophageal cancer models ($p = 2. \times 10^{-3}$ [breast], $p = 2.6 \times 10^{-3}$ [esophageal]; Figure 3E). In both osteosarcoma ($p = 1.4 \times 10^{-3}$) and lung cancer models ($p = 3.5 \times 10^{-2}$; Figure S1C), we identified an association between mutation/deletion of *CDKN2A* and dependency upon the cyclin dependent kinase gene *CDK11A*, which encodes a CDKN2A interacting protein (Varjosalo et al., 2013). In total, we identified 63 kinase dependencies associated with driver gene mutation status that were observed independently in more than one histotype (Table S1K).

## Integrating Data on Protein-Protein and Regulatory Interactions Facilitates the Interpretation of Genetic Dependencies

The set of KGDs associated with cancer driver gene alterations can be used to frame testable hypotheses, such as "mutation in gene *A* drives dependency upon a gene *B*." However, without further information, there are a number of potential mechanistic explanations for each genetic dependency. In model organisms, the problem of interpreting such dependencies has been addressed by integrating information from protein-protein (Beyer et al., 2007) and kinase-substrate interaction databases (Fiedler et al., 2009). To facilitate a mechanistic understanding of KGDs and to provide additional guidance for the design of subsequent experiments, we annotated our list of KGDs according to whether they involved known protein-protein interactions (Chatr-Aryamontri et al., 2015; Das and Yu, 2012), known kinase-substrate relationships (Lachmann and Ma'ayan, 2009), or known regulatory relationships (Cerami et al., 2011) between the driver gene and the identified dependency (see Experimental Procedures). Doing this, we found 113 KGDs involved pairs of genes with a previously reported functional relationship between the mutated driver gene and kinase target (Tables S1I and S1K). For example, mutation/amplification of *EGFR* in lung cancer cell lines was associated with an increased dependency upon FES ($p = 3 \times 10^{-2}$; Figure 3F), previously identified as an EGFR binding partner (Jones et al., 2006). Similarly, in esophageal cancer models, we identified a significant association between mutation of the chromatin remodeling factor gene *SMARCA4* and dependency upon the bromodomain protein BRD4 ($p = 6 \times 10^{-3}$; Figure 3F), previously identified as a protein interaction partner of SMARCA4 (Rahman et al., 2011). Among the dependencies associated with a kinase-substrate interaction, we found that mutation of *STK11* (*LKB1*) in ovarian cancer models was associated with an increased dependency upon *MARK2* ($p = 2 \times 10^{-3}$; Figure 3G), an LKB1 substrate (Lizcano et al., 2004). Similarly, we found that *MYC* (*cMYC*) amplified esophageal models had an increased dependency upon *MAPK1* (ERK-2, $p = 1.2 \times 10^{-2}$; Figure 3G), which is known to phosphorylate and stabilize the cMYC protein (Sears et al., 2000). We also identified a series of dependencies between cancer driver genes and their transcriptional targets, the majority of which focused upon *MYC*. In lung cancer models, we found that *MYC* amplification was associated with an increased dependency upon *CDKL5* ($5.6 \times 10^{-3}$; Figure 3H), a gene whose expression is regulated by *MYC*. Similarly, in esophageal models, we found *MYC* amplification to be associated with an increased dependency upon the *MYC* transcriptional target *PRKCH* (Zeller et al., 2006) ($p = 6.7 \times 10^{-3}$; Figure 3H).

For KGDs where a direct relationship between the driver gene and the kinase was not known, we used a simple information-flow type analysis to identify the shortest known molecular paths between driver gene and the kinase dependency (Tables S1I and S1K). For example, one of the strongest dependencies identified across all histotypes was between *STK11* and *SRP72* (Figure S4C). We found no evidence of a direct relationship between the two genes, but found that *STK11* has been shown to regulate the expression of *MYC* (Nath-Sain and Marignani, 2009), which in turn has been shown to regulate *SRP72* (Zeller et al., 2006), suggesting a putative path linking the driver gene and the kinase dependency. In esophageal cancer models, we found that *ERBB2* amplification is associated with *MASTL* (Voets and Wolthuis, 2010) and *NEK9* (Belham et al., 2003) KGDs (Figure 3I). We found no direct link between ERBB2 and either of these kinases, but both are CDK1 substrates and CDK1 itself is an ERBB2 substrate. In this instance, all members of the path (*ERBB2/CDK1/NEK9/MASTL*) were identified as *ERBB2* dependencies. In total, 163 dependencies not supported by a direct link could be reached by adding one intermediate connection (e.g., CDK1 is an intermediate connection between ERBB2 and NEK9).

## Pathway and Network Level KGDs

Work in model organisms has shown that a genetic mutation often results in an increased dependency on not just one gene, but multiple genes involved in a specific pathway or complex (Collins et al., 2007; Kelley and Ideker, 2005; Ryan et al., 2012). To explore the utility of this concept in interpreting our KGD data, we mapped the nominally significant KGDs ($p \leq 0.05$) identified for each cancer driver gene across all histotypes onto the high-confidence STRING functional interaction network (Franceschini et al., 2013) (see Experimental Procedures). For 11 of the 21 driver genes analyzed (*KRAS*, *ERBB2*, *CCND1*, *PIK3CA*, *SMAD4*, *NOTCH2*, *ARID1A*, *NF1*, *FBXW7*, *MAP2K4*, and *RB1*), we found that the dependencies associated with each driver gene were significantly more connected on the STRING interaction network than would be expected by chance (see Experimental Procedures; Figure S5; Table S1L). This suggested that these 11 driver genes might induce dependencies not just on individual genes, but on functional subnetworks. For two of these networks, we added known protein-protein and kinase-substrate interaction data to aid their interpretation. In the case of the network associated with *ERBB2* amplification, this suggested that *ERBB2* amplification might induce dependencies on direct binding partners and substrates of ERBB2 (JAK2, ERBB3, and PIK3CA), but also a network of genes involved in MAPK signaling (e.g., *MAP2K3*, *MAP3K4*, and *MAP3K2*) and inositol phosphate metabolism (including *PIP5K1A*, *PIK3CA*, and *PIK3CD*) (Figure 4A). Similarly, we found significantly more functional interactions among the kinases identified as dependencies associated with mutation of the tumor suppressor *SMAD4*, a member of the TGF-β pathway that

OPEN ACCESS
CellPress

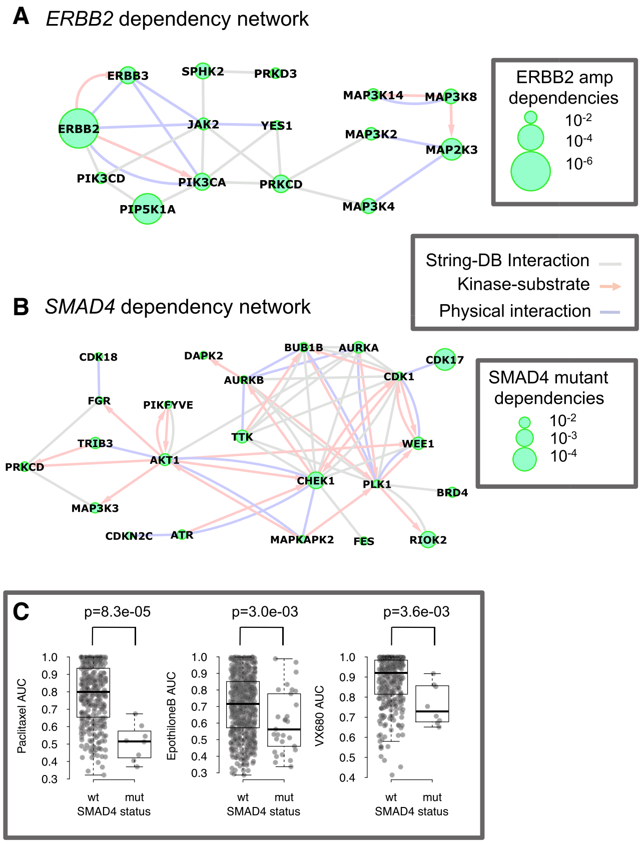


**Figure 4. Driver Gene KGDs and Functional Interaction Networks**

(A) Functional interaction network showing interactions between *ERBB2* amplification-associated KGDs. The nodes correspond to kinases that are identified as KGDs in *ERBB2* amplified cell lines. The nodes are scaled to indicate the significance of the KGD association p value. The blue edges correspond to experimentally determined protein-protein interactions, the pink arrows indicate the direction of experimentally determined kinase-substrate interactions, and the gray edges reflect high-confidence STRING functional interactions. Only KGDs that interact with at least one other *ERBB2* dependency are shown.

(B) Functional interaction network showing interactions among KGDs identified in *SMAD4* mutated cancer cell lines. Details as for *ERBB2* network in (A).

(C) Box plot showing AUC values of a panel of cell lines exposed to compounds targeting microtubules (paclitaxel and epothilone B) or Aurora Kinases (VX680) and classified into *SMAD4* mutant or wild-type groups. The top and bottom of the box represents the third and first quartiles and the box band represents the median (second quartile); whiskers extend to 1.5 times the interquartile distance from the box.

See also Figure S5 and Table S1L.

is frequently mutated or homozygously deleted in colorectal (Thiagalingam et al., 1996), pancreatic (Hahn et al., 1996), and esophageal (Dulak et al., 2013) cancers (Figure 4B). The integrated network we constructed from *SMAD4* KGDs revealed that *AKT1* and a number of its substrates (*FGR*, *MAP3K3*, *PIKFYVE*, *CHEK1*, and *WEE1*) were *SMAD4* mutation associated KGDs. Consistent with this, loss of *SMAD4* has been shown to be associated with increased AKT activation in colorectal and pancreatic tumor cell lines (Chen et al., 2014; Zhang et al., 2014). Furthermore, a recent large-scale drug screen identified *SMAD4* as the only driver gene significantly associated with sensitivity to A-443654, a pan-AKT inhibitor (Garnett et al., 2012). In addition to *AKT1* and its substrates, we found a densely connected group of kinases that regulate the mitotic cell cycle in the *SMAD4* dependency network (Figure 4B), suggesting that *SMAD4* mutant tumor cell lines may have an increased sensitivity to perturbation of this process. To test this hypothesis, we analyzed a compendium of drug sensitivity profiles (Garnett et al., 2012) and found that *SMAD4* mutant cell lines have increased sensitivity to the Aurora Kinase inhibitor VX-680 (Harrington et al., 2004) ($p = 4 \times 10^{-3}$; Figure 4C). Furthermore, we found that *SMAD4* mutant cell lines also exhibited an increased sensitivity to the mitotic inhibitors paclitaxel ($p = 8.3 \times 10^{-5}$) and epothilone B ($p = 3 \times 10^{-3}$; Figure 4C), suggesting a general sensitivity to drugs that target the mitotic checkpoint.

We present the functional interaction networks for the dependencies associated with each driver gene in Figure S5 and Table S1L. In addition to aiding the interpretation of dependencies, these subnetworks may be useful in alleviating some of the problems associated with false-positive effects in high-throughput genetic screens. Although there is a possibility of any given dependency being the result of off-target siRNA effects (Jackson and Linsley, 2010), the likelihood of an entire pathway being identified through off-target effects is likely to be much lower.

In the examples described above, we used the siRNA data to identify KGDs associated with defects in individual driver genes. Although there are hundreds of reported driver genes in cancer, some of these can be grouped into a small number of recurrently altered pathways (Garraway and Lander, 2013). Furthermore, it is possible that mutation in any member of such a pathway might have similar phenotypic effects. With this in mind, we considered whether we could identify candidate “pathway level” dependencies by grouping tumor cell lines according to mutations in any one of a set of driver genes belonging to the same pathway or complex. We obtained a previously curated list of pathways associated with driver gene mutations (Garraway and Lander, 2013) and manually updated this using literature information on well-established pathways (e.g., homologous recombination). For each pathway, tumor cell lines were grouped using a logical OR argument, i.e., if a cell line possessed a functional mutation of any gene member of the pathway then that cell line was considered mutated in that pathway. This resulted in a set of 15 pathway groupings (Table S1M) that were perturbed in at least seven tumor cell lines. Associating pathway mutations with KGDs was then performed in the same way as for individual genes using the MP test approach. This resulted in the identification of an additional 338 dependencies across all histotypes (Table S1N) and 748 histotype-specific dependencies (Table S1O).

As with individual driver genes, we found that the mutation of pathways was often associated with dependencies that were densely connected on the STRING functional interaction network. Indeed, using the dependencies identified across all histotypes, we found that nine of the 15 pathways (HR, PRC2, PI3K signaling, Cell Cycle Oncogenes, Cell Cycle Merged, TOR Signaling, MAPK Signaling, TGF B Signaling, and RAS/RAF Signaling) were associated with dependencies that were more functionally connected than would be expected by chance.

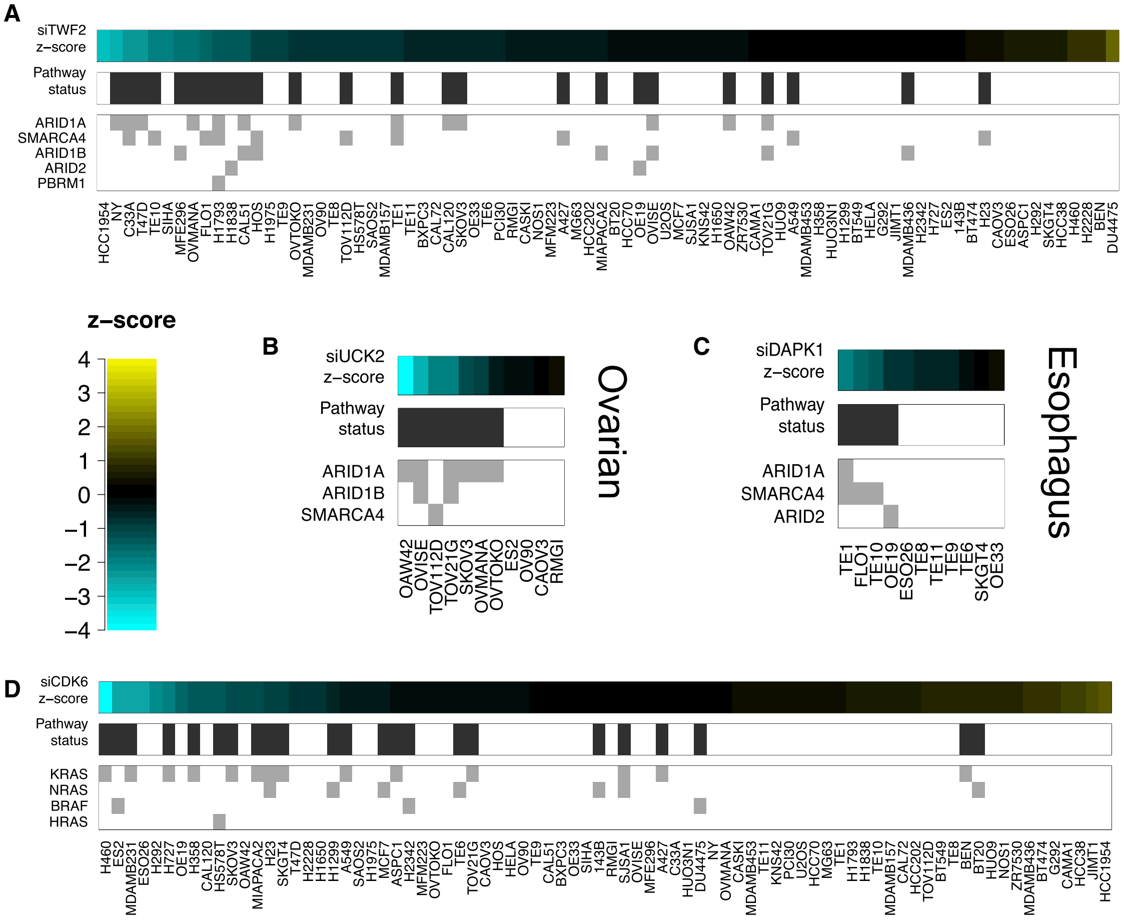


**Figure 5. Pathway Mutations Associated with KGDs**

(A) Heatmap showing increased dependency on *TWF2* in cell lines with loss-of-function mutations in members of the SWI-SNF complex.
(B) Heatmap showing increased dependency on *UCK2* in ovarian cancer cell lines with loss-of-function mutations in members of the SWI-SNF complex.
(C) Heatmap showing increased dependency on *DAPK1* in esophageal cancer cell lines with loss-of-function mutations in members of the SWI-SNF complex.
(D) Heatmap showing increased dependency upon *CDK6* in cell lines bearing mutations in *KRAS*, *HRAS*, *NRAS*, or *BRAF*.
See also Figure S5 and Tables S1M–S1O.

This suggested that mutation of one pathway may induce dependency on a second pathway, consistent with observations from yeast where it has been shown genetic dependencies can often be best explained as occurring between pairs of pathways (Kelley and Ideker, 2005). The dependency graphs associated with each pathway are presented in Figure S5.

In some instances, the association between a pathway and kinase siRNA had a predictive value no greater than the association with an individual member of the pathway. For example, alteration in the mTOR signaling pathway (mutation in *TSC1* OR *TSC2* OR *STK11*) was associated with an increased dependency upon the signal recognition particle *SRP72* gene (rho = −0.40), but mutation of *STK11* alone better explained the relative sensitivity of mutant and non-mutant cell lines in this regard (rho = −0.44). We therefore filtered these associations to identify 175 across-histotype (Table S1N) and 608 histotype-specific pathway dependencies where the pathway was a better predictor of dependency than any one individual gene (Table S1O). One example of a pathway dependency involved loss-of-function mutations in the genes encoding components of the SWI/SNF complex, mutated in ∼20% of all human cancers. By grouping all tumor cell lines that had a loss-of-function mutation or homozygous deletion of any member of the SWI/SNF complex (including the genes *ARID1A*, *SMARCA1*, *SMARCA4*, *ARID2*, *ARID1B*, and *PBRM1*) and then carrying out MP tests on the siRNA data as before, we identified ten KGDs including *TWF2* (Figure 5A), a gene encoding a protein that affects the stability of the actin cytoskeleton through interaction with G-actin (Pivovarova et al., 2013). Further dependencies were identified for this complex within specific histotypes including the uridine-cytidine kinase gene *UCK2* (Van Rompay et al., 2001) in ovarian cancer models (Figure 5B) and the death-associated protein kinase gene *DAPK1* in esophageal cancer models (Figure 5C).

We also investigated MAPK gene alterations (including *RAS* gene or *BRAF* mutations) as a pathway and found a *CDK6* KGD (Figure 5D). The dependency of *KRAS* mutant tumor cell lines upon CDK6 was readily apparent (rho = −0.38), but was stronger when *KRAS*, *HRAS*, *NRAS*, or *BRAF* mutant tumor cell line

models were combined as a group (rho = −0.43). CDKs, including CDK6, have been identified by a number of groups as putative non-oncogene addictions for *KRAS* mutant cancers (Barbie et al., 2009; Puyol et al., 2010). Our results suggest that *CDK6* might be a non-oncogene addiction not just for *KRAS* mutant models, but also for cell lines with any one of a variety of MAPK activating mutations (*NRAS*, *HRAS*, and *BRAF*). We tested this hypothesis using published drug screening results for a CDK4/6 inhibitor (PD0332991) in 628 cell lines (Garnett et al., 2012) and found a significant association between mutation of the MAPK pathway and sensitivity to this inhibitor ($p = 2.5 \times 10^{-3}$, Mann-Whitney U [MW U]-test). None of the individual members of this pathway showed as strong an association with this inhibitor (KRAS $p = 1.9 \times 10^{-1}$, HRAS $p = 5.8 \times 10^{-2}$, NRAS $p = 1.7 \times 10^{-2}$, BRAF $p = 2.8 \times 10^{-2}$, and MW U-test).

## DISCUSSION

A key challenge in the study of cancer biology is to understand how driver mutations alter the cellular state to promote tumor progression and how these altered states may be exploited in the development of targeted therapeutic approaches to the disease (Yaffe, 2013). Here, we have used siRNA screening to quantitatively estimate the kinase requirements of tumor cell lines in an attempt to understand better the genetic dependencies present. By integrating our siRNA data with molecular and histotype classifications, we have identified KGDs associated with particular cancer histologies or the presence of particular driver gene mutations. By integrating the KGD data with additional sources of annotation, such as protein-protein interaction data, we have tried to exemplify how testable hypotheses can be framed to explain the associations between a biomarker, such as a driver gene mutation, and a kinase dependency. Our aim in providing this data and illustrating its potential utility is to present starting points for further work.

As with any large functional data set, it is important to point out where elements of the technology used might influence the interpretation of the data. In general, siRNA mediated gene silencing is transient, when compared to, for example, short hairpin (sh) RNA mediated RNAi. With this in mind, we used a relatively short cell culture period between transfection and cell viability assessment (a 4 to 7 day period). Nevertheless, we cannot predict whether longer-term cell culture or longer-term gene silencing might result in a somewhat different profile of genetic dependencies. Furthermore, we used an ATP-based assay of cell viability in the screens. Some modes of cell inhibition exist that might have been missed using this method. As with any high-throughput technique, siRNA screens also have inherent false-positive and false-negative effects. Addressing false positives is especially important given the well-documented off-target effects associated with RNAi reagents (Jackson and Linsley, 2010). Consequently, we recommend that subsequent work that builds on the dependencies we have identified encapsulates some form of orthogonal validation. Individual siRNAs designed to target a gene (as we have shown in the case of *DYRK1A* dependency in *RB1* null cell lines) or small molecule inhibitors (as we have shown for the FGFR sensitivity of osteosarcoma cell lines) might be used as a form of validation. Alternatively, methods such as CRISPR-Cas9 mediated gene targeting (Sander and Joung, 2014) might be appropriate. We also note that like all genetic screen data sets, the negative predictive value of our data (i.e., the prediction that a particular genetic dependency does not exist) might be somewhat limited, given the transient and sometimes incomplete nature of gene silencing by siRNA.

In carrying out functional screens in cancer cell lines, we have tried to use some of the lessons learned from studies in model organisms to aid the interpretation of our identified dependencies. For example, integrating protein-protein interaction data with functional data (Figure 5) was an approach pioneered in the study of yeast genetic interaction screens (Beyer et al., 2007; Kelley and Ideker, 2005). Here, we have integrated this type of data to help frame testable hypotheses relating to the observed dependencies. A more sophisticated level of protein-protein interaction data for human tumor cell lines (Krogan et al., 2015) will undoubtedly enhance our ability to understand genetic dependencies. Similarly the availability of phosphoproteomic data for the cell lines in our panel may facilitate a more mechanistic reconstruction of the signaling networks active in each cell line. A number of approaches (e.g., So et al., 2015; Terfve and Saez-Rodriguez, 2012) have been developed to integrate siRNA or small molecule perturbations with time-course phosphoproteomics data sets to reconstruct signaling networks. Currently phosphoproteomic data for cancer cell line panels are relatively limited (e.g., Casado et al., 2013; Creixell et al., 2015), but as the overlap of cell lines covered by these phosphoproteomic resources and our siRNA resource increases there will be opportunities for the development of further integrative modeling approaches. Similarly, the increased availability of protein expression data sets (e.g., Lawrence et al., 2015; Moghaddas Gholami et al., 2013) may provide further opportunities for the development of additional integrative approaches.

Finally, to make our resource as useful to the community as possible, we have made all of the data described in this manuscript available (https://cansar.icr.ac.uk/), alongside the computational scripts used to integrate data (https://github.com/GeneFunctionTeam/cell_line_functional_annotation).

## EXPERIMENTAL PROCEDURES

### siRNA and Small Molecule Screening

Cell lines were transfected with a plate-arrayed siRNA library targeting 714 kinases and kinase-related genes (Dharmacon SMARTpools). Positive control (siPLK1) and multiple negative controls (siCON1 and siCON2; Dharmacon, catalog numbers D-001210-01-20 and D-001206-14-20) and AllStar (QIAGEN, catalog number 1027281) were included on every plate. 20 breast cancer models were screened in a 96-well-plate format while the remaining cell lines were screened in a 384-well-plate format (Table S1A). All screens were performed in triplicate. Cell viability was estimated as cells reached 70% confluency (normally 4–7 days after transfection) using a CellTiter-Glo assay (Promega). Data processing and quality control was performed using the cellHTS2 R package (Boutros et al., 2006). Further details, including small molecule sensitivity testing, are provided in the Supplemental Information.

### Association Testing

To identify associations between specific features (histotype or driver gene mutation) and sensitivity to specific siRNAs, a one-sided MP test was used. For each siRNA, we compared the observed difference between the median

*Z* score of the interest group and the median *Z* score of the "other" group to that expected based on random permutation. There were one million random samples that were created with the same sample sizes as the interest and other groups and the difference in the medians of the two groups calculated, allowing an empirically determined p value to be calculated. Correction for multiple testing was performed using the Benjamini and Hochberg FDR (Benjamini and Hochberg, 1995) and only those at an FDR of 50% are reported. For all small molecule association tests, we used a one-sided MW U test on area under the dose response curve values.

### Data Access

All siRNA *Z* score data can be found in Table S1B and also at https://cansar.icr.ac.uk/.

### Data Integration

Data from HINT (Das and Yu, 2012), BioGRID version 3.4.128 (Chatr-Aryamontri et al., 2015), and KEA protein- protein interaction databases were used (Lachmann and Ma'ayan, 2009). Kinase-substrate interactions were obtained from KEA (Lachmann and Ma'ayan, 2009), PhosphoSitePlus (Hornbeck et al., 2015) and (Cheng et al., 2014). High confidence (combined score >0.7) functional interactions were obtained from the STRING database (Version 9.1; Franceschini et al., 2013). Gene expression relationships were obtained from Pathway Commons (Cerami et al., 2011). The shortest_path function in NetworkX (Hagberg et al., 2008) was also used. Further details are provided in the Supplemental Information.

## ACCESSION NUMBERS

The accession number for the exome sequencing data of 11 ovarian cancer cell lines reported in this study is ENA: PRJEB9639.

## SUPPLEMENTAL INFORMATION

Supplemental Information includes Supplemental Experimental Procedures, five figures, and one table and can be found with this article online at http://dx.doi.org/10.1016/j.celrep.2016.02.023.

## AUTHOR CONTRIBUTIONS

C.J.L., A.A., C.J.R., and J.C. designed the experiments and wrote the manuscript. J.C. and C.J.R. performed statistical analyses and data integration. H.H., H.N.P., I.B., I.Y.C., J.F., R.B., R.M., R.R., S.C.-C., and S.P.-V. performed siRNA screens. C.T.W., H.H., H.N.P., and R.R. performed drug profiling. H.H. and R.B. performed protein quantitation. H.N.P. performed RT quantitative (q) PCR. A.G., J.C., and W.W. processed the siRNA and small molecule inhibitor screen data. J.T., B.A.-L., R.N., S.J.S., T.F., and D.A.Q. contributed materials, reagents, and analysis tools. A.A. and C.J.L. secured funding. All authors read and approved the final manuscript.

## ACKNOWLEDGMENTS

We thank Ultan McDermott from the Wellcome Trust Sanger Institute for sharing DNA sequencing data prior to publication. This work was funded by Cancer Research UK (grant number C347/A8363), Breast Cancer Now, UCSF, and The EU FP7 project EurocanPlatform (grant number 260791). C.J.R. is a Sir Henry Wellcome Fellow jointly funded by Science Foundation Ireland, the Health Research Board, and the Wellcome Trust (grant number 103049/Z/13/Z) under the SFI-HRB-Wellcome Trust Biomedical Research Partnership. We acknowledge National Health Service (NHS) funding to the National Institute for Health Research (NIHR) Royal Marsden Hospital Biomedical Research Centre.

Received: July 31, 2015
Revised: November 7, 2015
Accepted: February 1, 2016
Published: March 3, 2016

## REFERENCES

Bang, Y.J., Van Cutsem, E., Feyereislova, A., Chung, H.C., Shen, L., Sawaki, A., Lordick, F., Ohtsu, A., Omuro, Y., Satoh, T., et al.; ToGA Trial Investigators (2010). Trastuzumab in combination with chemotherapy versus chemotherapy alone for treatment of HER2-positive advanced gastric or gastro-oesophageal junction cancer (ToGA): a phase 3, open-label, randomised controlled trial. Lancet *376*, 687–697.

Bansal, R., Magge, S., and Winkler, S. (2003). Specific inhibitor of FGF receptor signaling: FGF-2-mediated effects on proliferation, differentiation, and MAPK activation are inhibited by PD173074 in oligodendrocyte-lineage cells. J. Neurosci. Res. *74*, 486–493.

Barbie, D.A., Tamayo, P., Boehm, J.S., Kim, S.Y., Moody, S.E., Dunn, I.F., Schinzel, A.C., Sandy, P., Meylan, E., Scholl, C., et al. (2009). Systematic RNA interference reveals that oncogenic KRAS-driven cancers require TBK1. Nature *462*, 108–112.

Barretina, J., Caponigro, G., Stransky, N., Venkatesan, K., Margolin, A.A., Kim, S., Wilson, C.J., Lehár, J., Kryukov, G.V., Sonkin, D., et al. (2012). The cancer cell line encyclopedia enables predictive modelling of anticancer drug sensitivity. Nature *483*, 603–607.

Belham, C., Roig, J., Caldwell, J.A., Aoyama, Y., Kemp, B.E., Comb, M., and Avruch, J. (2003). A mitotic cascade of NIMA family kinases. Nercc1/Nek9 activates the Nek6 and Nek7 kinases. J. Biol. Chem. *278*, 34897–34909.

Benjamini, Y., and Hochberg, Y. (1995). Controlling the false discovery rate: A practical and powerful approach to multiple testing. J. R. Stat. Soc., B *57*, 289–300.

Berriz, G.F., Beaver, J.E., Cenik, C., Tasan, M., and Roth, F.P. (2009). Next generation software for functional trend analysis. Bioinformatics *25*, 3043–3044.

Beyer, A., Bandyopadhyay, S., and Ideker, T. (2007). Integrating physical and genetic maps: from genomes to interaction networks. Nat. Rev. Genet. *8*, 699–710.

Boutros, M., Brás, L.P., and Huber, W. (2006). Analysis of cell-based RNAi screens. Genome Biol. *7*, R66.

Brough, R., Frankum, J.R., Sims, D., Mackay, A., Mendes-Pereira, A.M., Bajrami, I., Costa-Cabral, S., Rafiq, R., Ahmad, A.S., Cerone, M.A., et al. (2011). Functional viability profiles of breast cancer. Cancer Discov. *1*, 260–273.

Casado, P., Alcolea, M.P., Iorio, F., Rodríguez-Prados, J.C., Vanhaesebroeck, B., Saez-Rodriguez, J., Joel, S., and Cutillas, P.R. (2013). Phosphoproteomics data classify hematological cancer cell lines according to tumor type and sensitivity to kinase inhibitors. Genome Biol. *14*, R37.

Cerami, E.G., Gross, B.E., Demir, E., Rodchenkov, I., Babur, O., Anwar, N., Schultz, N., Bader, G.D., and Sander, C. (2011). Pathway Commons, a web resource for biological pathway data. Nucleic Acids Res. *39*, D685–D690.

Chatr-Aryamontri, A., Breitkreutz, B.J., Oughtred, R., Boucher, L., Heinicke, S., Chen, D., Stark, C., Breitkreutz, A., Kolas, N., O'Donnell, L., et al. (2015). The BioGRID interaction database: 2015 update. Nucleic Acids Res. *43*, D470–D478.

Chen, Y.W., Hsiao, P.J., Weng, C.C., Kuo, K.K., Kuo, T.L., Wu, D.C., Hung, W.C., and Cheng, K.H. (2014). SMAD4 loss triggers the phenotypic changes of pancreatic ductal adenocarcinoma cells. BMC Cancer *14*, 181.

Cheng, F., Jia, P., Wang, Q., and Zhao, Z. (2014). Quantitative network mapping of the human kinome interactome reveals new clues for rational kinase inhibitor discovery and individualized cancer therapy. Oncotarget *5*, 3697–3710.

Collins, S.R., Miller, K.M., Maas, N.L., Roguev, A., Fillingham, J., Chu, C.S., Schuldiner, M., Gebbia, M., Recht, J., Shales, M., et al. (2007). Functional dissection of protein complexes involved in yeast chromosome biology using a genetic interaction map. Nature *446*, 806–810.

Cowley, G.S., Weir, B.A., Vazquez, F., Tamayo, P., Scott, J.A., Rusin, S., East-Seletsky, A., Ali, L.D., Gerath, W.F.J., Pantel, S.E., et al. (2014). Parallel genome-scale loss of function screens in 216 cancer cell lines for the identification of context-specific genetic dependencies. Sci. Data *1*, 140035.

Creixell, P., Schoof, E.M., Simpson, C.D., Longden, J., Miller, C.J., Lou, H.J., Perryman, L., Cox, T.R., Zivanovic, N., Palmeri, A., et al. (2015). Kinome-wide decoding of network-attacking mutations rewiring cancer signaling. Cell *163*, 202–217.

Das, J., and Yu, H. (2012). HINT: High-quality protein interactomes and their applications in understanding human disease. BMC Syst. Biol. *6*, 92.

Dérijard, B., Raingeaud, J., Barrett, T., Wu, I.H., Han, J., Ulevitch, R.J., and Davis, R.J. (1995). Independent human MAP-kinase signal transduction pathways defined by MEK and MKK isoforms. Science *267*, 682–685.

Dulak, A.M., Stojanov, P., Peng, S., Lawrence, M.S., Fox, C., Stewart, C., Bandla, S., Imamura, Y., Schumacher, S.E., Shefler, E., et al. (2013). Exome and whole-genome sequencing of esophageal adenocarcinoma identifies recurrent driver events and mutational complexity. Nat. Genet. *45*, 478–486.

Fiedler, D., Braberg, H., Mehta, M., Chechik, G., Cagney, G., Mukherjee, P., Silva, A.C., Shales, M., Collins, S.R., van Wageningen, S., et al. (2009). Functional organization of the S. cerevisiae phosphorylation network. Cell *136*, 952–963.

Forbes, S.A., Beare, D., Gunasekaran, P., Leung, K., Bindal, N., Boutselakis, H., Ding, M., Bamford, S., Cole, C., Ward, S., et al. (2015). COSMIC: exploring the world's knowledge of somatic mutations in human cancer. Nucleic Acids Res. *43*, D805–D811.

Franceschini, A., Szklarczyk, D., Frankild, S., Kuhn, M., Simonovic, M., Roth, A., Lin, J., Minguez, P., Bork, P., von Mering, C., and Jensen, L.J. (2013). STRING v9.1: protein-protein interaction networks, with increased coverage and integration. Nucleic Acids Res. *41*, D808–D815.

Futreal, P.A., Coin, L., Marshall, M., Down, T., Hubbard, T., Wooster, R., Rahman, N., and Stratton, M.R. (2004). A census of human cancer genes. Nat. Rev. Cancer *4*, 177–183.

Garnett, M.J., Edelman, E.J., Heidorn, S.J., Greenman, C.D., Dastur, A., Lau, K.W., Greninger, P., Thompson, I.R., Luo, X., Soares, J., et al. (2012). Systematic identification of genomic markers of drug sensitivity in cancer cells. Nature *483*, 570–575.

Garraway, L.A., and Lander, E.S. (2013). Lessons from the cancer genome. Cell *153*, 17–37.

Gavine, P.R., Mooney, L., Kilgour, E., Thomas, A.P., Al-Kadhimi, K., Beck, S., Rooney, C., Coleman, T., Baker, D., Mellor, M.J., et al. (2012). AZD4547: an orally bioavailable, potent, and selective inhibitor of the fibroblast growth factor receptor tyrosine kinase family. Cancer Res. *72*, 2045–2056.

Greenman, C., Stephens, P., Smith, R., Dalgliesh, G.L., Hunter, C., Bignell, G., Davies, H., Teague, J., Butler, A., Stevens, C., et al. (2007). Patterns of somatic mutation in human cancer genomes. Nature *446*, 153–158.

Grueneberg, D.A., Li, W., Davies, J.E., Sawyer, J., Pearlberg, J., and Harlow, E. (2008). Kinase requirements in human cells: IV. Differential kinase requirements in cervical and renal human tumor cell lines. Proc. Natl. Acad. Sci. USA *105*, 16490–16495.

Hagberg, A., Schult, D., and Swart, P. (2008). Exploring network structure, dynamics, and function using NetworkX. G. Varoquaux, T. Vaught, and J. Millman, eds. Proceedings of the 7th Python in Science Conference, 11–16.

Hahn, S.A., Schutte, M., Hoque, A.T., Moskaluk, C.A., da Costa, L.T., Rozenblum, E., Weinstein, C.L., Fischer, A., Yeo, C.J., Hruban, R.H., and Kern, S.E. (1996). DPC4, a candidate tumor suppressor gene at human chromosome 18q21.1. Science *271*, 350–353.

Harrington, E.A., Bebbington, D., Moore, J., Rasmussen, R.K., Ajose-Adeogun, A.O., Nakayama, T., Graham, J.A., Demur, C., Hercend, T., Diu-Hercend, A., et al. (2004). VX-680, a potent and selective small-molecule inhibitor of the Aurora kinases, suppresses tumor growth in vivo. Nat. Med. *10*, 262–267.

Hornbeck, P.V., Zhang, B., Murray, B., Kornhauser, J.M., Latham, V., and Skrzypek, E. (2015). PhosphoSitePlus, 2014: mutations, PTMs and recalibrations. Nucleic Acids Res. *43*, D512–D520.

Jackson, A.L., and Linsley, P.S. (2010). Recognizing and avoiding siRNA off-target effects for target identification and therapeutic application. Nat. Rev. Drug Discov. *9*, 57–67.

Jones, R.B., Gordus, A., Krall, J.A., and MacBeath, G. (2006). A quantitative protein interaction network for the ErbB receptors using protein microarrays. Nature *439*, 168–174.

Kaelin, W.G., Jr. (2005). The concept of synthetic lethality in the context of anticancer therapy. Nat. Rev. Cancer *5*, 689–698.

Kansara, M., Teng, M.W., Smyth, M.J., and Thomas, D.M. (2014). Translational biology of osteosarcoma. Nat. Rev. Cancer *14*, 722–735.

Kelley, R., and Ideker, T. (2005). Systematic interpretation of genetic interactions using protein networks. Nat. Biotechnol. *23*, 561–566.

Koh, J.L., Brown, K.R., Sayad, A., Kasimer, D., Ketela, T., and Moffat, J. (2012). COLT-Cancer: functional genetic screening resource for essential genes in human cancer cell lines. Nucleic Acids Res. *40*, D957–D963.

Krogan, N.J., Lippman, S., Agard, D.A., Ashworth, A., and Ideker, T. (2015). The cancer cell map initiative: defining the hallmark networks of cancer. Mol. Cell *58*, 690–698.

Lachmann, A., and Ma'ayan, A. (2009). KEA: kinase enrichment analysis. Bioinformatics *25*, 684–686.

Lawrence, R.T., Perez, E.M., Hernández, D., Miller, C.P., Haas, K.M., Irie, H.Y., Lee, S.I., Blau, C.A., and Villén, J. (2015). The proteomic landscape of triple-negative breast cancer. Cell Rep. *11*, 630–644.

Lizcano, J.M., Göransson, O., Toth, R., Deak, M., Morrice, N.A., Boudeau, J., Hawley, S.A., Udd, L., Mäkelä, T.P., Hardie, D.G., and Alessi, D.R. (2004). LKB1 is a master kinase that activates 13 kinases of the AMPK subfamily, including MARK/PAR-1. EMBO J. *23*, 833–843.

Loijens, J.C., and Anderson, R.A. (1996). Type I phosphatidylinositol-4-phosphate 5-kinases are distinct members of this novel lipid kinase family. J. Biol. Chem. *271*, 32937–32943.

Lord, C.J., Tutt, A.N., and Ashworth, A. (2015). Synthetic lethality and cancer therapy: lessons learned from the development of PARP inhibitors. Annu. Rev. Med. *66*, 455–470.

Luo, J., Solimini, N.L., and Elledge, S.J. (2009). Principles of cancer therapy: oncogene and non-oncogene addiction. Cell *136*, 823–837.

Madaule, P., Eda, M., Watanabe, N., Fujisawa, K., Matsuoka, T., Bito, H., Ishizaki, T., and Narumiya, S. (1998). Role of citron kinase as a target of the small GTPase Rho in cytokinesis. Nature *394*, 491–494.

Manning, G., Whyte, D.B., Martinez, R., Hunter, T., and Sudarsanam, S. (2002). The protein kinase complement of the human genome. Science *298*, 1912–1934.

Marcotte, R., Brown, K.R., Suarez, F., Sayad, A., Karamboulas, K., Krzyzanowski, P.M., Sircoulomb, F., Medrano, M., Fedyshyn, Y., Koh, J.L., et al. (2012). Essential gene profiles in breast, pancreatic, and ovarian cancer cells. Cancer Discov. *2*, 172–189.

Miller, T.W., Rexer, B.N., Garrett, J.T., and Arteaga, C.L. (2011). Mutations in the phosphatidylinositol 3-kinase pathway: role in tumor progression and therapeutic implications in breast cancer. Breast Cancer Res. *13*, 224.

Moghaddas Gholami, A., Hahne, H., Wu, Z., Auer, F.J., Meng, C., Wilhelm, M., and Kuster, B. (2013). Global proteome analysis of the NCI-60 cell line panel. Cell Rep. *4*, 609–620.

Nath-Sain, S., and Marignani, P.A. (2009). LKB1 catalytic activity contributes to estrogen receptor alpha signaling. Mol. Biol. Cell *20*, 2785–2795.

Pivovarova, A.V., Chebotareva, N.A., Kremneva, E.V., Lappalainen, P., and Levitsky, D.I. (2013). Effects of actin-binding proteins on the thermal stability of monomeric actin. Biochemistry *52*, 152–160.

Prahallad, A., Sun, C., Huang, S., Di Nicolantonio, F., Salazar, R., Zecchin, D., Beijersbergen, R.L., Bardelli, A., and Bernards, R. (2012). Unresponsiveness of colon cancer to BRAF(V600E) inhibition through feedback activation of EGFR. Nature *483*, 100–103.

Puyol, M., Martín, A., Dubus, P., Mulero, F., Pizcueta, P., Khan, G., Guerra, C., Santamaría, D., and Barbacid, M. (2010). A synthetic lethal interaction between K-Ras oncogenes and Cdk4 unveils a therapeutic strategy for non-small cell lung carcinoma. Cancer Cell *18*, 63–73.

Rahman, S., Sowa, M.E., Ottinger, M., Smith, J.A., Shi, Y., Harper, J.W., and Howley, P.M. (2011). The Brd4 extraterminal domain confers transcription activation independent of pTEFb by recruiting multiple proteins, including NSD3. Mol. Cell. Biol. *31*, 2641–2652.

Ryan, C.J., Roguev, A., Patrick, K., Xu, J., Jahari, H., Tong, Z., Beltrao, P., Shales, M., Qu, H., Collins, S.R., et al. (2012). Hierarchical modularity and the evolution of genetic interactomes across species. Mol. Cell *46*, 691–704.

Sadasivam, S., and DeCaprio, J.A. (2013). The DREAM complex: master coordinator of cell cycle-dependent gene expression. Nat. Rev. Cancer *13*, 585–595.

Sakharkar, M.K., and Sakharkar, K.R. (2007). Targetability of human disease genes. Curr. Drug Discov. Technol. *4*, 48–58.

Sander, J.D., and Joung, J.K. (2014). CRISPR-Cas systems for editing, regulating, and targeting genomes. Nat. Biotechnol. *32*, 347–355.

Sears, R., Nuckolls, F., Haura, E., Taya, Y., Tamai, K., and Nevins, J.R. (2000). Multiple Ras-dependent phosphorylation pathways regulate Myc protein stability. Genes Dev. *14*, 2501–2514.

So, J., Pasculescu, A., Dai, A.Y., Williton, K., James, A., Nguyen, V., Creixell, P., Schoof, E.M., Sinclair, J., Barrios-Rodiles, M., et al. (2015). Integrative analysis of kinase networks in TRAIL-induced apoptosis provides a source of potential targets for combination therapy. Sci. Signal. *8*, rs3.

Su, N., Du, X., and Chen, L. (2008). FGF signaling: its role in bone development and human skeleton diseases. Front. Biosci. *13*, 2842–2865.

Terfve, C., and Saez-Rodriguez, J. (2012). Modeling signaling networks using high-throughput phospho-proteomics. Adv. Exp. Med. Biol. *736*, 19–57.

Thiagalingam, S., Lengauer, C., Leach, F.S., Schutte, M., Hahn, S.A., Overhauser, J., Willson, J.K., Markowitz, S., Hamilton, S.R., Kern, S.E., et al. (1996). Evaluation of candidate tumour suppressor genes on chromosome 18 in colorectal cancers. Nat. Genet. *13*, 343–346.

Tischler, J., Lehner, B., and Fraser, A.G. (2008). Evolutionary plasticity of genetic interaction networks. Nat. Genet. *40*, 390–391.

Van Rompay, A.R., Norda, A., Lindén, K., Johansson, M., and Karlsson, A. (2001). Phosphorylation of uridine and cytidine nucleoside analogs by two human uridine-cytidine kinases. Mol. Pharmacol. *59*, 1181–1186.

Varjosalo, M., Keskitalo, S., Van Drogen, A., Nurkkala, H., Vichalkovski, A., Aebersold, R., and Gstaiger, M. (2013). The protein interaction landscape of the human CMGC kinase group. Cell Rep. *3*, 1306–1320.

Voets, E., and Wolthuis, R.M. (2010). MASTL is the human orthologue of Greatwall kinase that facilitates mitotic entry, anaphase, and cytokinesis. Cell Cycle *9*, 3591–3601.

Vogelstein, B., Papadopoulos, N., Velculescu, V.E., Zhou, S., Diaz, L.A., Jr., and Kinzler, K.W. (2013). Cancer genome landscapes. Science *339*, 1546–1558.

Workman, P., and Al-Lazikani, B. (2013). Drugging cancer genomes. Nat. Rev. Drug Discov. *12*, 889–890.

Yaffe, M.B. (2013). The scientific drunk and the lamppost: massive sequencing efforts in cancer discovery and treatment. Sci. Signal. *6*, pe13.

Zeller, K.I., Zhao, X., Lee, C.W., Chiu, K.P., Yao, F., Yustein, J.T., Ooi, H.S., Orlov, Y.L., Shahab, A., Yong, H.C., et al. (2006). Global mapping of c-Myc binding sites and target gene networks in human B cells. Proc. Natl. Acad. Sci. USA *103*, 17834–17839.

Zhang, J., Yang, P.L., and Gray, N.S. (2009). Targeting cancer with small molecule kinase inhibitors. Nat. Rev. Cancer *9*, 28–39.

Zhang, B., Zhang, B., Chen, X., Bae, S., Singh, K., Washington, M.K., and Datta, P.K. (2014). Loss of Smad4 in colorectal cancer induces resistance to 5-fluorouracil through activating Akt pathway. Br. J. Cancer *110*, 946–957.

Cell Reports, Volume *14*

## Supplemental Information

# Large-Scale Profiling of Kinase Dependencies

# in Cancer Cell Lines

**James Campbell, Colm J. Ryan, Rachel Brough, Ilirjana Bajrami, Helen N. Pemberton, Irene Y. Chong, Sara Costa-Cabral, Jessica Frankum, Aditi Gulati, Harriet Holme, Rowan Miller, Sophie Postel-Vinay, Rumana Rafiq, Wenbin Wei, Chris T. Williamson, David A. Quigley, Joe Tym, Bissan Al-Lazikani, Timothy Fenton, Rachael Natrajan, Sandra J. Strauss, Alan Ashworth, and Christopher J. Lord**

**SUPPLEMENTAL FIGURES**

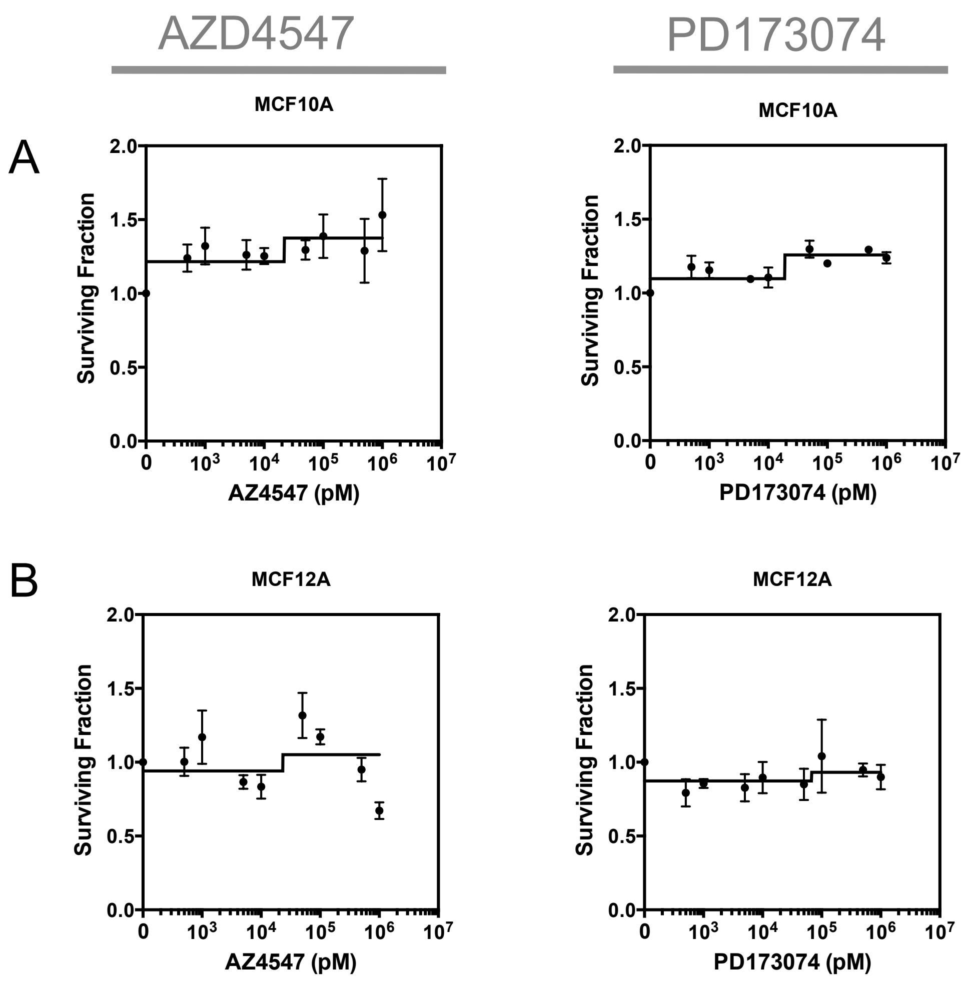


**Figure S1. Response to two small molecule FGFR inhibitors (AZD4547 and PD173074) in non-tumour derived epithelial cell lines. (Related to Figure 2).** A) Response of the normal epithelium-derived cell line MCF10A to eight concentrations of AZD4547 and PD173074. B) Response of the normal epithelium-derived cell line MCF12A to eight concentrations of AZD4547. Error bars represent standard error of the mean from three independent experiments.

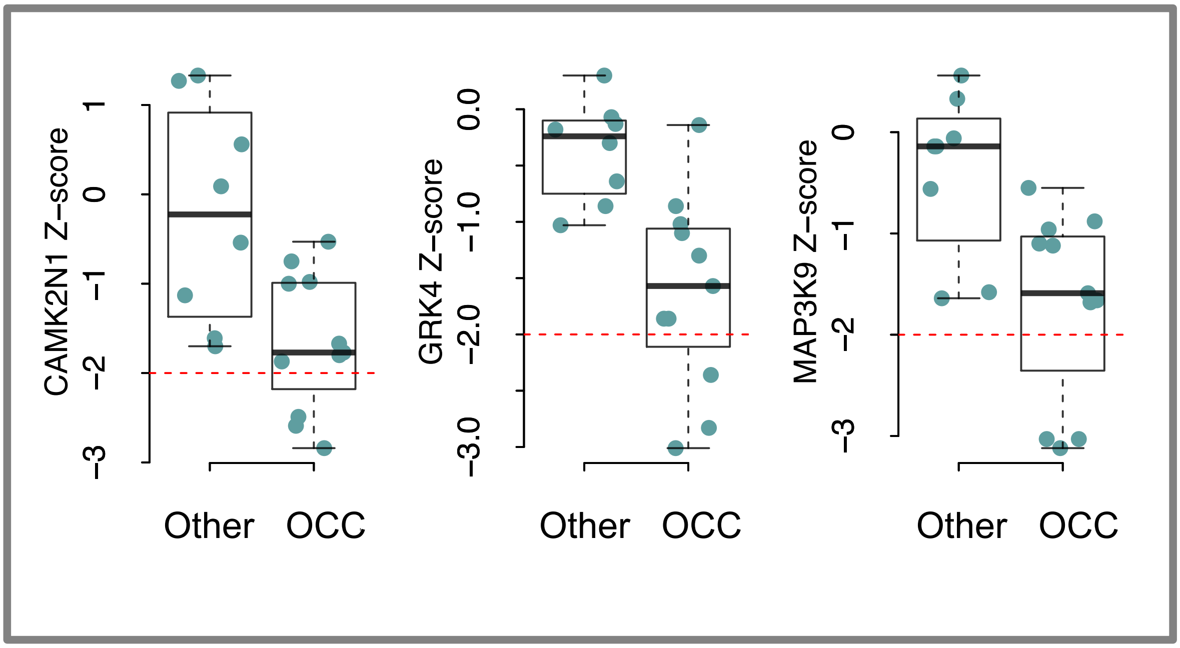


**Figure S2. Kinase dependencies associated with ovarian clear cell carcinoma. (Related to Figure 2).** Boxplots illustrating the dependency of 11 ovarian clear cell (OCC) models on *CAMK2N1*, *GRK4* and *MAP3K9* relative to ovarian carcinoma models classified as other subtypes. See also Supplementary Table S1F. In each box plot the top and bottom of the box represents the third and first quartiles and the box band represents the median (second quartile); whiskers extend to 1.5 times the interquartile distance from the box.

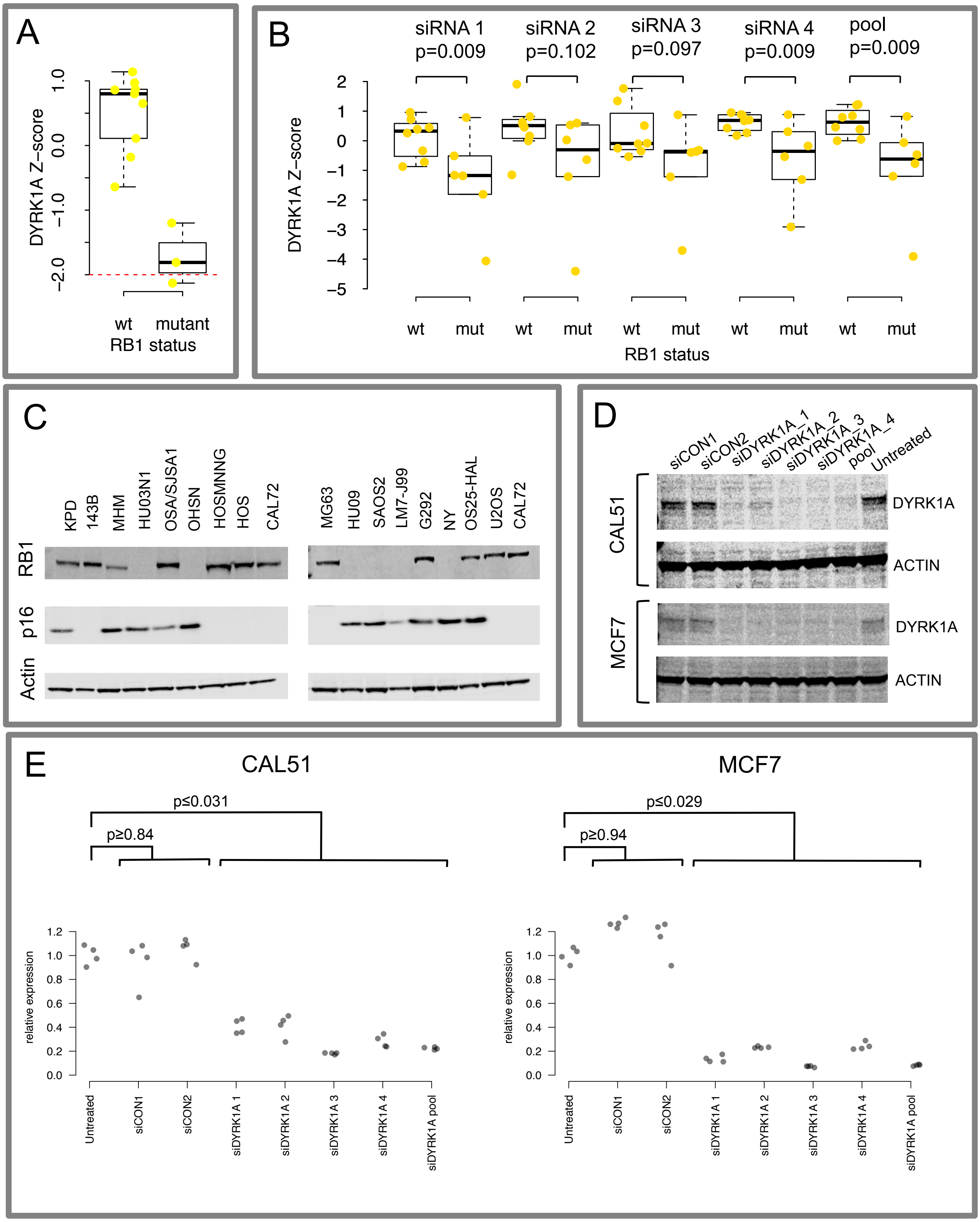


**Figure S3. Validation of DYRK1A dependency in RB1 mutant osteosarcoma cell lines. (Related to Figure 3).** A) Boxplot summarising Z-scores of osteosarcoma models treated with siRNAs targeting *DYRK1A* and grouped according to RB1 mutation status. B) *DYRK1A* siRNA smart pool deconvolution in *RB1* wt and mutant osteosarcoma models. P-values shown for each siRNA and the pool of siRNAs (siPool) are the result of applying median permutation tests to the siRNA Z-scores comparing the mutant and wild type groups. C) Detection of RB1, p16 (CDKN2A) and actin in osteosarcoma cell lines using western blotting and immunostaining of proteins. D) CAL51 and MCF7 cells treated with individual siRNAs targeting *DYRK1A* together with a pool of the same siRNAs, non-targeting negative control siRNAs (siCON1, siCON2) or untreated cells were harvested 72 h post transfection. DYRK1A and Actin protein abundance was evaluated using western blotting and immunostain detection. E) Expression of *DYRK1A vs GAPDH* mRNA in CAL51 and MCF7 cells treated with siRNAs. Relative expression values were normalized to the median of the untreated samples. Explanation as for D.

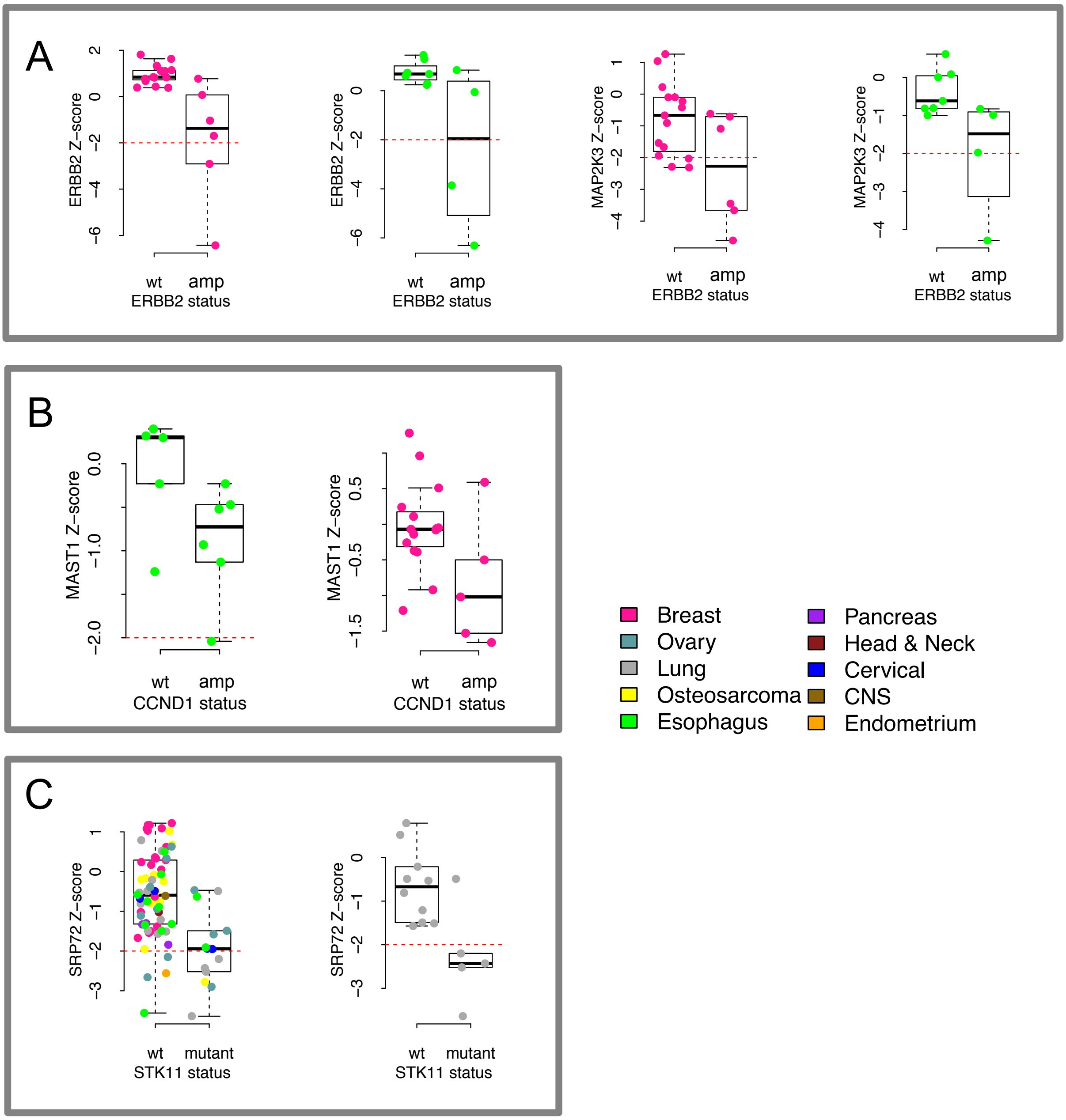


**Figure S4. Boxplots showing kinase dependencies associated with driver gene mutations. (Related to Figure 3).** A) Genetic dependencies associated with *ERBB2* amplification within breast and esophageal cancer histotypes. B) Genetic dependencies associated with *CCND1* amplification within breast and esophageal cancer histotypes. C) Dependency of *STK11* (*LKB1*) mutant cell lines to siRNA designed to target the Signal Recognition Particle 72 gene *SRP72* in the complete panel of cell lines and within the lung cancer panel. In each box plot the top and bottom of the box represents the third and first quartiles and the box band represents the median (second quartile); whiskers extend to 1.5 times the interquartile distance from the box.

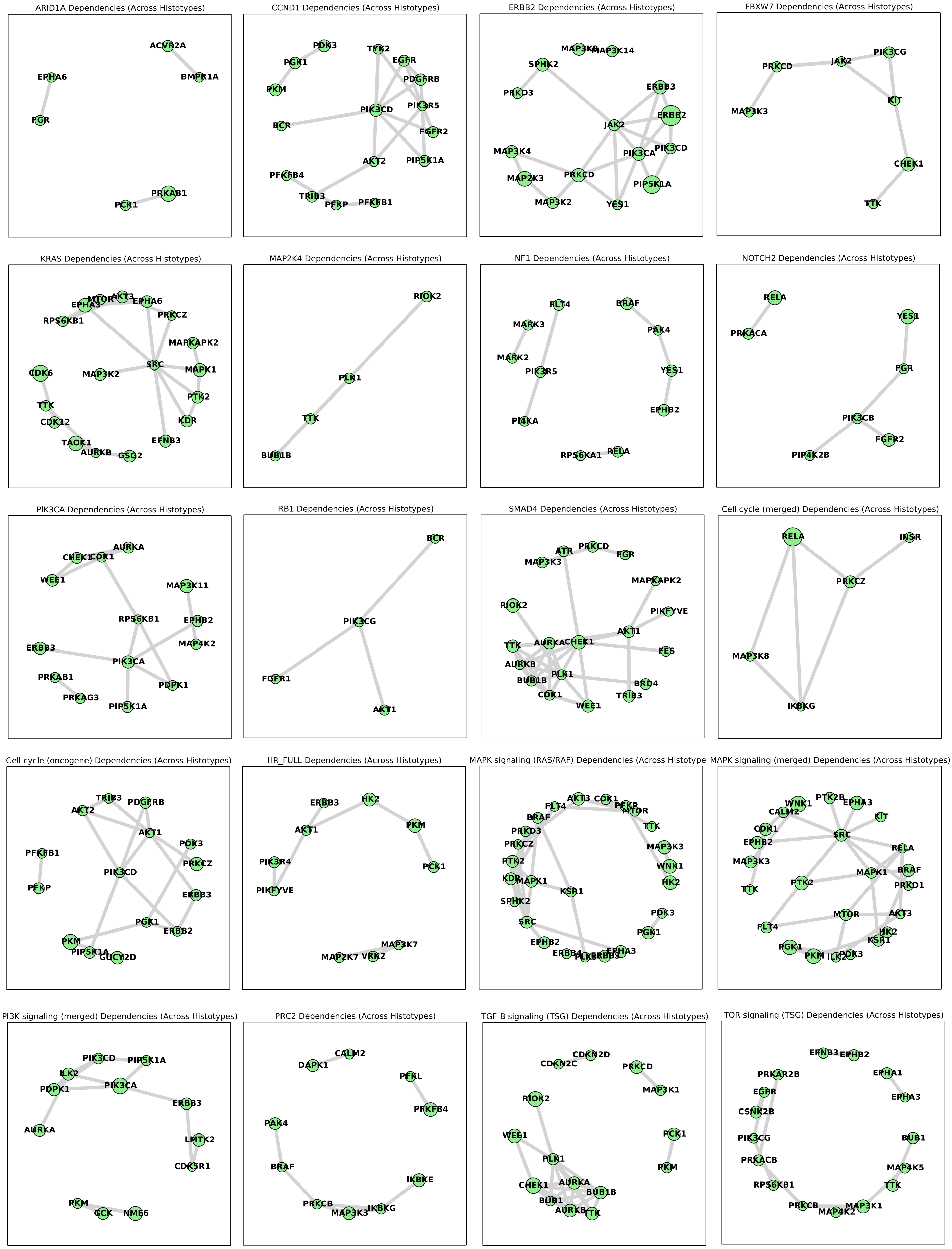


**Figure S5. Network dependency maps for driver genes and pathways. (Related to Figure 4 and Figure 5).** Functional interaction networks showing high-confidence STRING interactions between KGDs associated with 11 driver genes and 9 pathways. Only those networks that are more densely connected than expected are shown. See also Supplementary Table S1L

**Table S1.** Datasets used in the study and analysis result tables. The sheet named ‘explanation of the tables’ details the contents.

## Supplemental Experimental Procedures

### Cell lines and siRNA

The majority of tumor cell lines were obtained from the American Type Culture Collection, European Collection of Cell Cultures and Deutsche Sammlung von Mikroorganismen und Zellkulturen. Head and neck cancer cell line models were obtained from Susanne Gollin and Theresa Whiteside (University of Pittsburgh), Tom Carey (University of Michigan) and Hans Joenje (VU Medical Centre, NL). All cell lines were maintained as per the supplier's instructions. STR typing of 10 loci was performed on each cell line using the GenePrint 10 system (Promega) and used to confirm the identity of cell lines prior to storage. Cell lines were transfected with siRNA SMARTpools (Dharmacon) using Dharmafect 3, Dharmafect 4 (Dharmacon), Oligofectamine, Lipofectamine 2000 or RNAi max (Invitrogen) transfection reagents. siPLK1 (Dharmacon) was used as a positive control on each screen plate. Negative controls were siCON1, siCON2 (Dharmacon) and Allstar (Qiagen) and were included on every screening plate.

### siRNA screening and processing

136 cancer-derived cell lines spanning 10 different tissue types were optimised for siRNA screening. This involved titrating the amount of lipid transfection reagent used as well as titrating cell number used for reverse siRNA transfections. For each cell line we tested at least four different transfection reagents and selected conditions that met the following criteria: (i) compared to a mock control (no lipid or siRNA), the transfection of non-silencing negative control siRNA caused no more than 20 % cell inhibition; (ii) compared to non-silencing negative control siRNA, the transfection of PLK1–targeting siRNA caused more than 80% cell inhibition; (iii) cell confluency reached 70% within the range of 4-7 days. The later criteria allowed assays to be terminated whilst cells were in growth phase. Once optimal conditions had been established, each cell line was transfected with a plate-arrayed siRNA library targeting 714 kinases and kinase-related genes (Dharmacon). 20 breast cancer cell lines were screened in a 96 well plate format, while the remainder were screened in a 384 well plate format (Supplementary Table S1A). Cell viability was estimated using a luminescent assay detecting cellular ATP levels (CellTitre-Glo, Promega). Luminescence values were processed using the cellHTS2 R package (Boutros et al., 2006). To evaluate the effect of each siRNA pool on cell viability, we log2 transformed the luminescence measurements and then centred these to the median value for each plate. The plate-centred data were scaled to the median absolute deviation (MAD) of the library as a whole to produce robust Z-scores (Figure 1A). All screens were performed in triplicate. Screens judged to have poor dynamic range (Z' factor $\leq 0$) (Zhang et al., 1999) or poorly correlated replicates ($r \leq 0.7$) were excluded during an evaluation of screen quality. Subsequently, we retained kinase dependency profiles for 117 cell line models for further analysis.

### Small molecule kinase inhibitor cell inhibition assays

Cell lines were profiled using two FGFR inhibitors (AZD4547 and PD173074) at eight different concentrations (0.5, 1, 5, 10, 50, 100, 500 and 1000 nM). A majority of the panel of cell lines profiled were derived from breast cancer and osteosarcomas with smaller numbers of cell lines derived from tumors of the central nervous system, cervix, head and neck, large intestine and lung cancer. 250-500 cells were seeded in 384 well plates. 24 hours post seeding, drug exposure was initiated and cells were continuously cultured in the presence of the drugs for a period of five days, at which point cell viability was estimated using Cell-Titre Glo (Promega). Cells were initially plated at a density to ensure that each cell line was in growth phase by the end of the five day treatment, similar to (Garnett et al., 2012). Luminescence values were normalised to the median of the per-plate DMSO negative control wells and the dose-response relationships modelled using 3-parameter logistic regression provided by the drc R-package (Ritz and Streibig, 2005). Area under the dose response curve (AUC) measurements calculated using this package were used as a read-out of drug sensitivity. Each cell line was assessed in triplicate.

### Annotating cell lines according to mutation status

We annotated cell lines according to the presence of DNA mutations and copy number alterations in oncogenes and tumor suppressor genes. We included genes in the Cancer Gene Census (Futreal et al., 2004), genes listed in a census of amplified and overexpressed genes in human cancer (Santarius et al., 2010) and genes identified as candidate tumor suppressor gene (TSG) or oncogenes (OG) using the TUSON explorer approach described by (Davoli et al., 2013). The set of cancer genes were classified into

a tumor suppressor (TSG)-like group and an oncogene (OG)-like group using the molecular genetic classification information for genes available in the Cancer Gene Census. Genes with dominant effects were classified an oncogenes and those with recessive effects classified as tumor suppressor genes. Genes that were not included in the Cancer Gene Census were classified using the TUSON Explorer TSG and OG q-values (a positive classification occurring where the q-score for a given gene was < 0.1).

We obtained exome sequencing data for 77 cell lines from the COSMIC cell line project (http://cancer.sanger.ac.uk). We extended this data set with new exome sequencing data from 11 ovarian cancer cell lines (deposited at http://www.ebi.ac.uk/ena/data/view/PRJEB9639, exome pipeline described in a later section). We also obtained gene copy number data for 86 cell lines from the CCLE project (http://www.broadinstitute.org/ccle/) (Barretina et al., 2012). To annotate cell lines according to likely functionally mutated cancer genes we built a pipeline that takes mutation information from whole exome sequencing experiments and somatic copy number variation (CNVs) from multiple sources and classifies each cell line as to whether or not there is evidence for mutation or CNV for each of the cancer genes (https://github.com/GeneFunctionTeam/cell_line_functional_annotation). This annotation pipeline conforms to guidelines for the integration of functional genomics data proposed by the International Cancer Genome Consortium (Gonzalez-Perez et al., 2013). A dictionary of terms describing mutational consequences in the various data sets was used to enable comparison across each data set. Specifically, terms relating to copy number amplification, homozygous deletion, truncating mutations or recurrent missense mutations were used to define those mutations most likely to have a functional consequence in affected cell lines. Where a mutation was observed that was deemed to have an uncertain consequence (for example, a non-recurrent missense mutation), the event was recorded so that during the association tests any cell lines with such mutations in a gene could be excluded from both the mutant and wild-type group. The annotation pipeline also performed standardisation of cell line names and gene identifiers.

Using the exome data we considered a TSG-like gene to be functionally mutated in a given cell line if it contained a likely loss-of-function mutation (frameshift, nonsense or splice site alteration) or a missense mutation at a recurrently mutated residue (described in the next paragraph). In contrast we only considered an OG-like gene to be functionally mutated if it contained a mutation of a recurrently mutated residue. For CNV data sets we classified cell lines as containing a functionally relevant mutation if there was evidence of homozygous deletion of a TSG-like gene (GISTIC score of -2) or genomic amplification of an OG-like gene (GISTIC score of 2).

Even for well established cancer-associated genes, distinguishing between driver mutations (that have a likely gain of function effect for oncogenes or loss of function effect for tumor suppressors) and passenger mutations (that have no oncogenic effect) can be difficult for missense somatic mutations (point mutations). To address this problem we attempted to distinguish between the two by focussing on recurrent mutations - those mutations that alter residues that are frequently mutated across multiple tumor samples. We defined recurrently mutated residues using a database of > 1.2 million somatic mutations (Davoli et al., 2013). As some genes are sequenced more frequently than others (due to targeted sequencing) or mutated more frequently than others (due to genome location / chromatin accessibility) the threshold for defining a residue recurrently mutated is set on a per gene basis. For the analysis described here we defined a recurrently mutated residue as one that is mutated in either 3% of all samples featuring a mutation in that gene or three samples overall, whichever threshold is larger.

The set of functionally relevant alterations in cancer genes based on the exome and CNV data types were represented as Boolean matrices (1 for mutant, 0 for non-mutant). These datasets were combined using a simple logical OR function. For example, combining exome and CNV data sets we considered an oncogene to be mutated in a given cell line if the oncogene was either amplified according to the CNV data OR mutated at a recurrently altered site according to the exome data. Cell lines where a given data type was not available were excluded from analyses incorporating that data type.

**Exome sequencing pipeline**

Exome sequencing libraries were prepared using SureSelect Human All Exon 50 Mbp kits (Agilent). Illumina paired-end libraries were sequenced on a HiSeq2000 (Illumina), acquiring 2 x 76 bp reads. Basecalling and demultiplexing was performed using Casava v1.8 (Illumina) software. Fastq files were

aligned to the human reference genome (GRCh37) using the Burrows-Wheeler Aligner (Li and Durbin, 2009). Duplicate reads arising from PCR were removed prior to further processing and variant detection. Base recalibration, realignment around indels and variant calling were performed using the Genome Analysis Tool-kit v2 with default settings (DePristo et al., 2011). Variants called in regions not covered by the capture probes were excluded, as were those with genotype quality scores below 20 and those covered by fewer than 10 reads.

**Protein Quantitation**
Whole cell protein lysates were extracted from cells by lysis with NP250 buffer (20 mM Tris pH 7.6, 1 mM EDTA, 0.5%. NP40, 250 mM NaCl). For RNAi knock-down, samples were extracted 72 hours post transfection. Western blots were performed using Novex precast TA gels (Invitrogen) as described previously (Farmer et al, 2005). Primary antibodies were immunoblotted overnight using either anti-RB1 (1/1000; Cell Signaling), anti-DYRK1A (1/1000 Cell Signalling), anti-p16 (1/1000 Abcam) or anti-ACTIN (1/2000; Santa Cruz). Fluorescent anti-rabbit, anti-mouse or anti-goat secondary antibodies (Licor) were incubated with the blot (1:5000) for 1 hour at room temperature in the dark followed by detection and processing on a Licor Odyssey Western Imager. Blots were viewed using ImageStudio Software (Licor).

**Quantitative RT–PCR**
Total RNA from cell lines were extracted using RNeasy mini kit (Qiagen) according to the manufacturer's instructions. cDNA was synthesised using SuperScript III reverse transcriptase (Invitrogen) for RT–PCR with random hexamers as per the manufacturer's instructions. Assay-on-Demand primer/probe sets were purchased from Applied Biosystems. Real-Time qPCR was performed on the 7900 DHT Fast Real-Time PCR System (Applied Biosystems), using GAPDH as an endogenous control. Gene expression was calculated relative to expression of GAPDH endogenous control. Samples were quantified in quadruplicate.

**Data Integration**
Protein-protein interactions were obtained from the HINT (Das and Yu, 2012), BioGRID version 3.4.128 (Chatr-Aryamontri et al., 2015) and KEA databases (Lachmann and Ma'ayan, 2009). Kinase-substrate interactions were obtained from KEA (Lachmann and Ma'ayan, 2009), PhosphoSitePlus (Hornbeck et al., 2015) and (Cheng et al., 2014). High confidence (combined score > 0.7) functional interactions were obtained from the STRING database (Version 9.1; (Franceschini et al., 2013)). Gene expression relationships were obtained from PathwayCommons (Cerami et al., 2011) by extracting those relationships annotated as 'controls-expression-of'. To identify the shortest directed path between driver genes and their dependencies we built an integrated directed network using the directed edges from PathwayCommons (those labelled 'controls-expression-of', 'controls-phosphorylation-of', 'controls-transport-of', 'controls-production-of') and the full set of kinase-substrate interactions from all sources. The shortest_path function in NetworkX (Hagberg et al., 2008) was then used to query this graph. All shortest paths of length 2 or less are provided in Supplemental Table S1I and S1K. To evaluate the connectivity of the dependencies associated with each driver gene we compared the number of observed high-confidence STRING interactions between the nominally significant dependencies (those with median permutation test p-value < 0.05) to the number observed on 100 randomized degree matched interaction networks.

**Pathway analysis**
The original pathway groupings were taken from (Garraway and Lander, 2013). Four groupings were dropped because they only contain a single gene (e.g. the 'RNA abundance' pathway contained a single gene *DIS3*). Six groupings were removed because they referred to a broad molecular class of genes ('RTK Signalling', 'Chromatin histone methyltransferases') rather than a specific pathway or complex. Three further groupings were removed as they were catch-all terms for unclassified driver genes (e.g. 'Other signaling'). We then added three additional pathways to this list – homologous recombination (based on data described by Wood and Lindahl (Wood et al., 2005; Wood et al., 2001), FGFR signalling (containing the FGFR genes which were previously only annotated as 'RTK signaling') and the PRC2 complex (Kuzmichev et al., 2002). From this list we then removed groupings that were not represented by any mutant cell lines in our panel or groupings in which only a single member of the grouping was mutated in our cell lines. Finally pathways that were essentially redundant (comprised of identical sets of

mutated genes) in the panel of cell lines we screened were combined. The pathways used are presented in Table S1M.

**Supplemental references**

Barretina, J., Caponigro, G., Stransky, N., Venkatesan, K., Margolin, A.A., Kim, S., Wilson, C.J., Lehar, J., Kryukov, G.V., Sonkin, D., *et al.* (2012). The Cancer Cell Line Encyclopedia enables predictive modelling of anticancer drug sensitivity. Nature *483*, 603-607.

Boutros, M., Bras, L.P., and Huber, W. (2006). Analysis of cell-based RNAi screens. Genome Biol *7*, R66.

Cerami, E.G., Gross, B.E., Demir, E., Rodchenkov, I., Babur, O., Anwar, N., Schultz, N., Bader, G.D., and Sander, C. (2011). Pathway Commons, a web resource for biological pathway data. Nucleic Acids Res *39*, D685-690.

Chatr-Aryamontri, A., Breitkreutz, B.J., Oughtred, R., Boucher, L., Heinicke, S., Chen, D., Stark, C., Breitkreutz, A., Kolas, N., O'Donnell, L., *et al.* (2015). The BioGRID interaction database: 2015 update. Nucleic Acids Res *43*, D470-478.

Cheng, F., Jia, P., Wang, Q., and Zhao, Z. (2014). Quantitative network mapping of the human kinome interactome reveals new clues for rational kinase inhibitor discovery and individualized cancer therapy. Oncotarget *5*, 3697-3710.

Das, J., and Yu, H. (2012). HINT: High-quality protein interactomes and their applications in understanding human disease. BMC Syst Biol *6*, 92.

Davoli, T., Xu, A.W., Mengwasser, K.E., Sack, L.M., Yoon, J.C., Park, P.J., and Elledge, S.J. (2013). Cumulative haploinsufficiency and triplosensitivity drive aneuploidy patterns and shape the cancer genome. Cell *155*, 948-962.

DePristo, M.A., Banks, E., Poplin, R., Garimella, K.V., Maguire, J.R., Hartl, C., Philippakis, A.A., del Angel, G., Rivas, M.A., Hanna, M., *et al.* (2011). A framework for variation discovery and genotyping using next-generation DNA sequencing data. Nat Genet *43*, 491-498.

Franceschini, A., Szklarczyk, D., Frankild, S., Kuhn, M., Simonovic, M., Roth, A., Lin, J., Minguez, P., Bork, P., von Mering, C., *et al.* (2013). STRING v9.1: protein-protein interaction networks, with increased coverage and integration. Nucleic Acids Res *41*, D808-815.

Futreal, P.A., Coin, L., Marshall, M., Down, T., Hubbard, T., Wooster, R., Rahman, N., and Stratton, M.R. (2004). A census of human cancer genes. Nature Rev Cancer *4*, 177-183.

Garraway, L.A., and Lander, E.S. (2013). Lessons from the cancer genome. Cell *153*, 17-37.

Gonzalez-Perez, A., Mustonen, V., Reva, B., Ritchie, G.R., Creixell, P., Karchin, R., Vazquez, M., Fink, J.L., Kassahn, K.S., Pearson, J.V.*, et al.* (2013). Computational approaches to identify functional genetic variants in cancer genomes. Nat Methods *10*, 723-729.

Hagberg, A., Schult, D., and Swart, P. (2008). Exploring Network Structure, Dynamics, and Function using NetworkX. Proceedings of the 7th Python in Science Conference.

Hornbeck, P.V., Zhang, B., Murray, B., Kornhauser, J.M., Latham, V., and Skrzypek, E. (2015). PhosphoSitePlus, 2014: mutations, PTMs and recalibrations. Nucleic Acids Res *43*, D512-520.

Kuzmichev, A., Nishioka, K., Erdjument-Bromage, H., Tempst, P., and Reinberg, D. (2002). Histone methyltransferase activity associated with a human multiprotein complex containing the Enhancer of Zeste protein. Genes Dev *16*, 2893-2905.

Lachmann, A., and Ma'ayan, A. (2009). KEA: kinase enrichment analysis. Bioinformatics *25*, 684-686.

Li, H., and Durbin, R. (2009). Fast and accurate short read alignment with Burrows-Wheeler transform. Bioinformatics *25*, 1754-1760.

Ritz, C., and Streibig, J.C. (2005). Bioassay Analysis using R. J Statist Software *12*, 1-22.

Santarius, T., Shipley, J., Brewer, D., Stratton, M.R., and Cooper, C.S. (2010). A census of amplified and overexpressed human cancer genes. Nature Rev Cancer *10*, 59-64.

Wood, R.D., Mitchell, M., and Lindahl, T. (2005). Human DNA repair genes, 2005. Mutat Res *577*, 275-283.

Wood, R.D., Mitchell, M., Sgouros, J., and Lindahl, T. (2001). Human DNA repair genes. Science *291*, 1284-1289.

Zhang, J.H., Chung, T.D., and Oldenburg, K.R. (1999). A Simple Statistical Parameter for Use in Evaluation and Validation of High Throughput Screening Assays. J Biomol Screen *4*, 67-73.
